# Prevalence and patterns of antimicrobial resistance among wildlife populations in Africa: a systematic review

**DOI:** 10.1038/s44259-025-00179-z

**Published:** 2026-02-16

**Authors:** Jemimmah W. Mwangi, Anne Kimeu, Arshnee Moodley, Peter K. Koskei, Katharina Schaufler, Dishon M. Muloi

**Affiliations:** 1 Kenya Field Epidemiology and Laboratory Training Program (FELTP), Moi University, Eldoret, Kenya; 2 Health Program, International Livestock Research Institute (ILRI), Nairobi, Kenya; 3 Department of Veterinary and Animal Sciences, University of Copenhagen, Frederiksberg C, Denmark; 4 Moi University, School of Public Health, Department of Epidemiology and Medical Statistics, Eldoret, Kenya; 5 Epidemiology and Ecology of Antimicrobial Resistance (GEAR), Helmholtz Institute for One Health, Fleischmannstraße 42, 17489 Greifswald, Germany; 6 Institute of Infection, Veterinary and Ecological Sciences, University of Liverpool, Liverpool, UK

**Keywords:** Diseases, Ecology, Ecology, Microbiology

## Abstract

Antimicrobial resistance (AMR) is increasingly reported in wildlife, yet evidence from Africa remains fragmented. We conducted a systematic review of AMR in African wildlife, screening 4,802 records and including 61 studies from 21 countries. Phenotypic testing was performed in all studies, primarily using disk diffusion (82%), with genotypic assays in 86.8%. Across 4,669 bacterial isolates from 27 eligible studies, the pooled prevalence of phenotypic resistance was 59% (95% CI: 34–80%), with substantial heterogeneity (I² = 97.4%). Wild birds exhibited the highest pooled prevalence (93%), followed by non-human primates (35%) and herbivores (25%). *Escherichia coli* (20 studies and 3414 isolates) showed a pooled resistance prevalence of 62%. Pooled multidrug resistance was 23.1% (9 studies and 1128 isolates). Sampling was predominantly opportunistic and concentrated in human-impacted environments, limiting ecological inference. These findings highlight significant AMR occurrence across diverse wildlife taxa and substantial gaps in surveillance, coverage, and methodological consistency.

## Introduction

The rising prevalence of antimicrobial-resistant bacteria is recognised as a critical global health challenge, primarily driven by antibiotic use and misuse in both human and animal populations^1,2^. To date, most research on antimicrobial resistance (AMR) has been focused on clinical, environmental and agricultural settings, whereas wildlife remains an underexplored and overlooked cohort^3–5^. This is despite increasing evidence indicating that wildlife populations can serve as important reservoirs of resistant bacteria and may disseminate resistance determinants within the broader environment^6^. Wildlife, human and livestock populations are interconnected through multiple ecological and epidemiological pathways, such as rodents inhabiting households^7^, shared water sources and grazing areas between wildlife and livestock^8^, or via ecosystems contaminated by anthropogenic waste, which may serve as hotspots for resistance-gene acquisition by wildlife. This inextricable link is particularly pronounced in Africa, where humans and wildlife have more opportunities for extensive interactions against the backdrop of high wildlife diversity and variable antimicrobial stewardship practices in human and agricultural systems, which often contribute to environmental contamination and potential spillover pathways^9,10^.

AMR determinants of clinical significance, including extended-spectrum β-lactamase (ESBL) genes such as *bla*_*CTX-M*_*, bla*_*TEM*_ and *bla*_*SHV*_, carbapenemase genes (bla_OXA-48_, *bla*_*NDM*_, and *bla*_*KPC*_), plasmid-mediated colistin resistance genes (*mcr-1* to *mcr-9*), and methicillin resistance determinants (*mecA*, *mecC*), have been documented across a wide array of bacterial species (both commensal and zoonotic pathogens) across diverse wildlife taxa globally, as well as within Africa specifically^4,11–16^. These reports span from peri-domestic wildlife, such as rodents with limited home ranges, migratory bird populations with extensive geographical ranges, and even animals inhabiting relatively pristine and minimally disturbed natural ecosystems^17–20^. Within the African context, emerging research have described AMR profiles in African wildlife, but a comprehensive synthesis across bacterial species and regions is lacking.

Despite the growing evidence highlighting wildlife’s putative role in AMR transmission, most national AMR action plans across the continent, and globally, do not explicitly acknowledge wildlife as key reservoirs or targets for surveillance. In addition, few policies or surveillance programmes, if any, specifically target wildlife^21^. Recently, however, there has been a renewed emphasis on incorporating wildlife into AMR surveillance within a comprehensive One Health framework, underscoring the critical need for data to quantify the wildlife-associated AMR burden. Only a few meta-analyses on prevalence of AMR in wildlife have been done globally^11,22^, and most of these analyses have typically included few studies from Africa, limiting their applicability and the extent to which they can inform understanding of AMR transmission dynamics within the continent.

The present study systematically reviews publicly available data on AMR prevalence in African wildlife. Specifically, we aim to estimate the prevalence of AMR bacteria in African wildlife and examine variation across host species and bacterial species. These findings provide a foundation for future AMR research, surveillance, and policy efforts to mitigate AMR risks at the wildlife-livestock-human interface in Africa^21–23^.

## Results

### Study characteristics

**Fig. 1:**
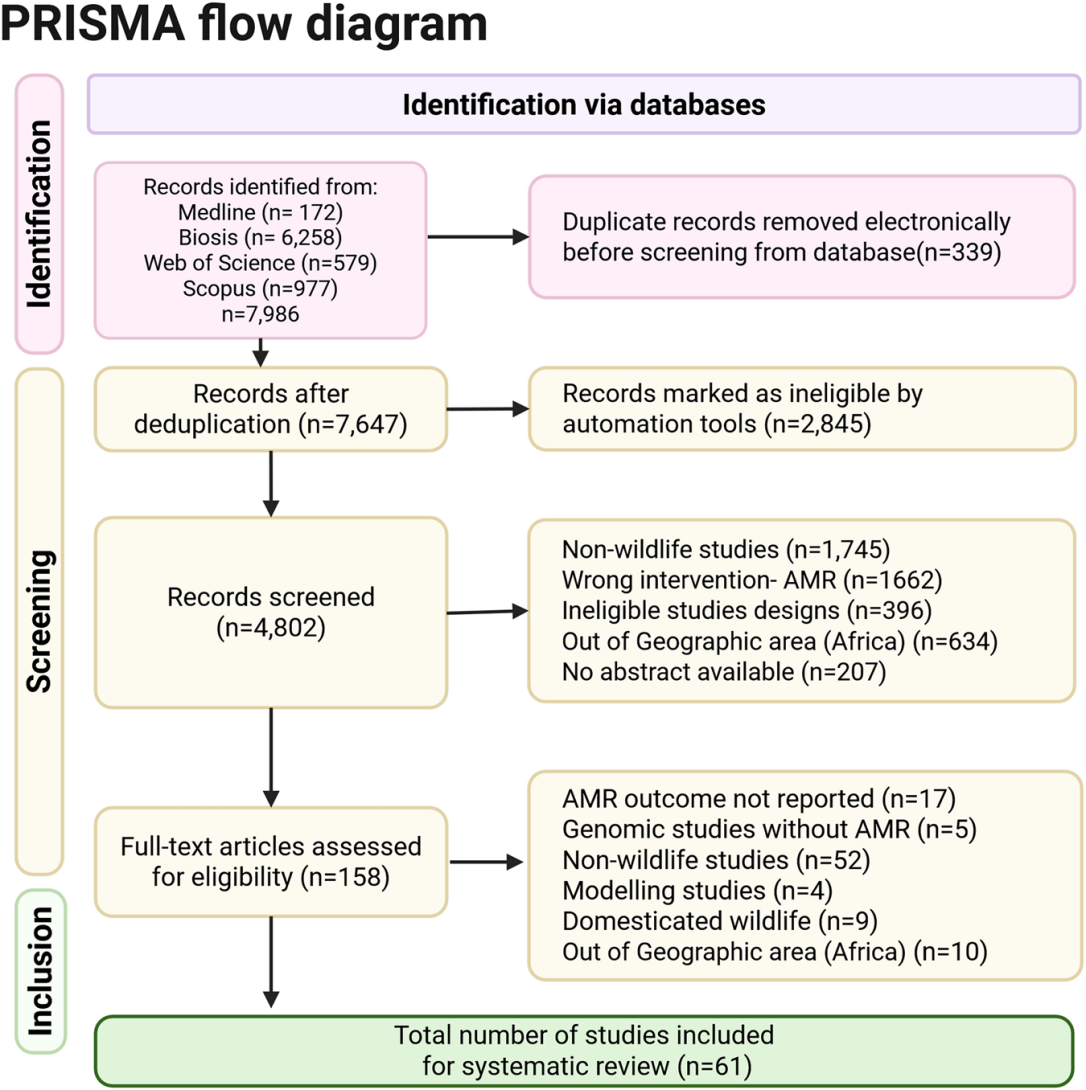
PRISMA flow diagram summarising study selection for the systematic review. Flow of information through the phases of identification, screening, eligibility assessment, and inclusion. A total of 4802 records were identified from four databases, of which 61 studies met the inclusion criteria. Figure created in https://BioRender.com.

The initial search identified 4802 articles. After reviewing titles and abstracts, 158 articles were selected for full-text screening, of which 61 fulfilled the inclusion criteria. We summarised key characteristics of the 61 studies included in the review, covering host species, bacterial taxa, habitat types, AMR outcomes, and testing methodologies (Fig. 1). Detailed study-level information, including sample sizes, resistance prevalence, sampling strategies, and interpretive standards is presented in Supplementary Table 2.

**Fig. 2:**
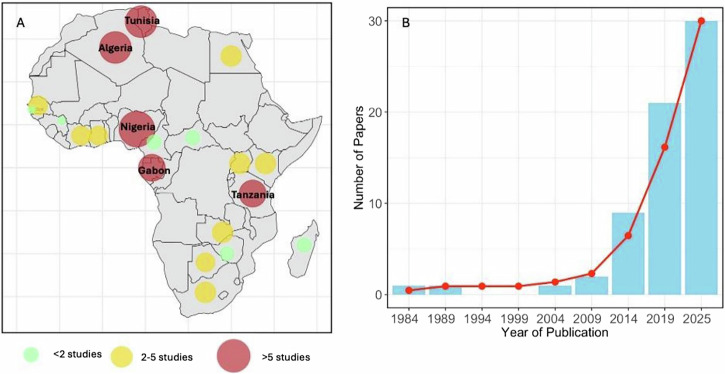
Geographic and temporal distribution of studies on antimicrobial resistance (AMR) in African wildlife. **A** Number of included studies for each African country, with darker shading indicating higher publication counts. **B** Annual trend in publications on AMR in wildlife across Africa from 1980 to 2025; the red line represents the cumulative total.

The 61 studies originated from 21 African countries, with most studies conducted in North and West Africa (30/61, 49.1%) and the fewest from Southern Africa (10/61, 16.4%). Specifically, Algeria, Gabon, and Nigeria accounted for the highest number of studies (21/61, 34.4%) (Fig. 2A). The studies were published between 1980 and 2024, with 88% (5/61) published between 2012 and 2023, reflecting increased interest in wildlife-associated AMR research in Africa (Fig. 2B). Sampling efforts predominantly occurred in conservation areas or locations close to human settlements, with opportunistic, non-probability sampling methods commonly employed. Only ten studies (*n* = 61, 16.4%) reported sampling wildlife populations from minimally disturbed or pristine habitats^14,15,24–31^.

All included studies used observational designs. Primary data collection was most common, occurring in 55 studies (90%), whereas six studies (10%) utilised previously collected samples, either exclusively (*n* = 3) or alongside new data collection (*n* = 3). Faecal samples were the predominant sample type, reported in 49 studies (80.3%), followed by necropsy-derived samples such as intestinal swabs, reported in eight studies (13%). Less common sample types included pharyngeal, oral, ear, or skin swabs, each reported in two studies (3%).

### Host populations and bacterial organisms isolated

**Fig. 3:**
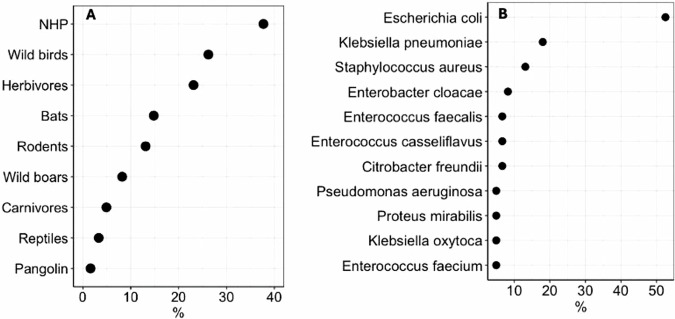
Distribution of wildlife host groups and bacterial species reported in AMR studies across Africa. **A** Proportion of included studies reporting antimicrobial resistance in each wildlife host group. **B** The top 11 bacterial species identified across studies. Percentages represent the proportion of total included studies in which each host or bacterial species was reported.

The reviewed studies included diverse wildlife species categorised into nine functional groups. Non-human primates (NHP) were the most frequently studied, appearing in 37.7% of studies^25,27–48^, (23/61) followed by wild birds (26.2%; 16 studies)^12,13,26,35,45,47,49–58^, herbivores (21.3%; 13 studies)^35,40,42,47,50,59–66^, and bats (14.8%; 9 studies)^14,24,45,67–72^. Rodents were represented in 13.1% (eight studies)^36,40,45,60,73–76^, wild boars in 8.2% (five studies)^15,28,47,77^, carnivores in 4.9% (three studies)^35,59,78^, and reptiles in 3.3% (two studies)^35,60^. Pangolins were the least studied group, appearing in just one study (1.6%)^79^ (Fig. 3a and Supplementary Table 4). Sample sizes varied widely, with 31% of studies (19/61) sampling 100–199 animals, 25% (15/61) sampling 50-99, and 21% (13/61) involving fewer than 50 animals. Nine studies (15%) included between 200 and 299 animals. Only six studies reported larger cohorts: two included 300–399 animals^32,39^, three studies had 400–700 animals^47,57,72^, and another had more than 700 animals^45^. In total, 55 unique bacterial species were identified. *Escherichia coli* was the most frequently reported organism, appearing in more than half of studies (52.5%, 32/61), followed by *Klebsiella pneumoniae* (18.0%, 11/61) and *Staphylococcus aureus* (13.1%, 8/61) (Fig. 3B).

Phenotypic antimicrobial susceptibility testing was the commonly used diagnostic method primarily performed using the disk diffusion method (82%, 50/61). Automated systems such as VITEK2 were used in 6.6% (four studies), and minimum inhibitory concentrations (MIC) were reported in another 6.6% (four studies). Genotypic testing was conducted in 86.8% (53/61) of studies to complement phenotypic methods, predominantly using Polymerase chain reaction (PCR) (86.7%, 46/53), with whole genome sequencing (WGS) performed in fewer studies (13%, 7/53).

A total of 81 unique antibiotics were assessed for phenotypic resistance across the 61 studies. Beta-lactam antibiotics were the most frequently tested class (tested in 51/61 studies, 83.6%) (e.g. ampicillin, amoxicillin-clavulanic acid, cefotaxime and ceftazidime), followed by Tetracyclines (primarily tetracycline) in 45/61 studies (73.8%). Both fluoroquinolones (primarily ciprofloxacin) and aminoglycosides (e.g. gentamicin and streptomycin) were each tested in 42/61 studies (68.9%). Phenicols (primarily chloramphenicol) were tested in 34/61 studies (55.7%) and antifolate agents, including trimethoprim alone or in combination with sulfamethoxazole, were tested in32/61 studies (52.5%). Less frequently tested classes included carbapenems (e.g. meropenem and imipenem) and macrolides (primarily erythromycin), each in 16/61 studies (26.2%), with lincosamides (e.g. clindamycin and lincomycin) being the least tested antibiotics (7/61 studies, 11.5%). Quantitative synthesis of resistance by antibiotic class was not feasible because most studies did not report class-specific resistance proportions with corresponding numerators and denominators, and testing panels varied widely across studies. To avoid analytical bias, we therefore summarised only the frequency of antibiotic classes tested and described dominant resistance patterns qualitatively. Supplementary Table 7 illustrates the heterogeneous reporting formats and inconsistent antibiotic coverage that precluded class-level meta-analysis.

### Overall prevalence of antibiotic resistance

**Fig. 4:**
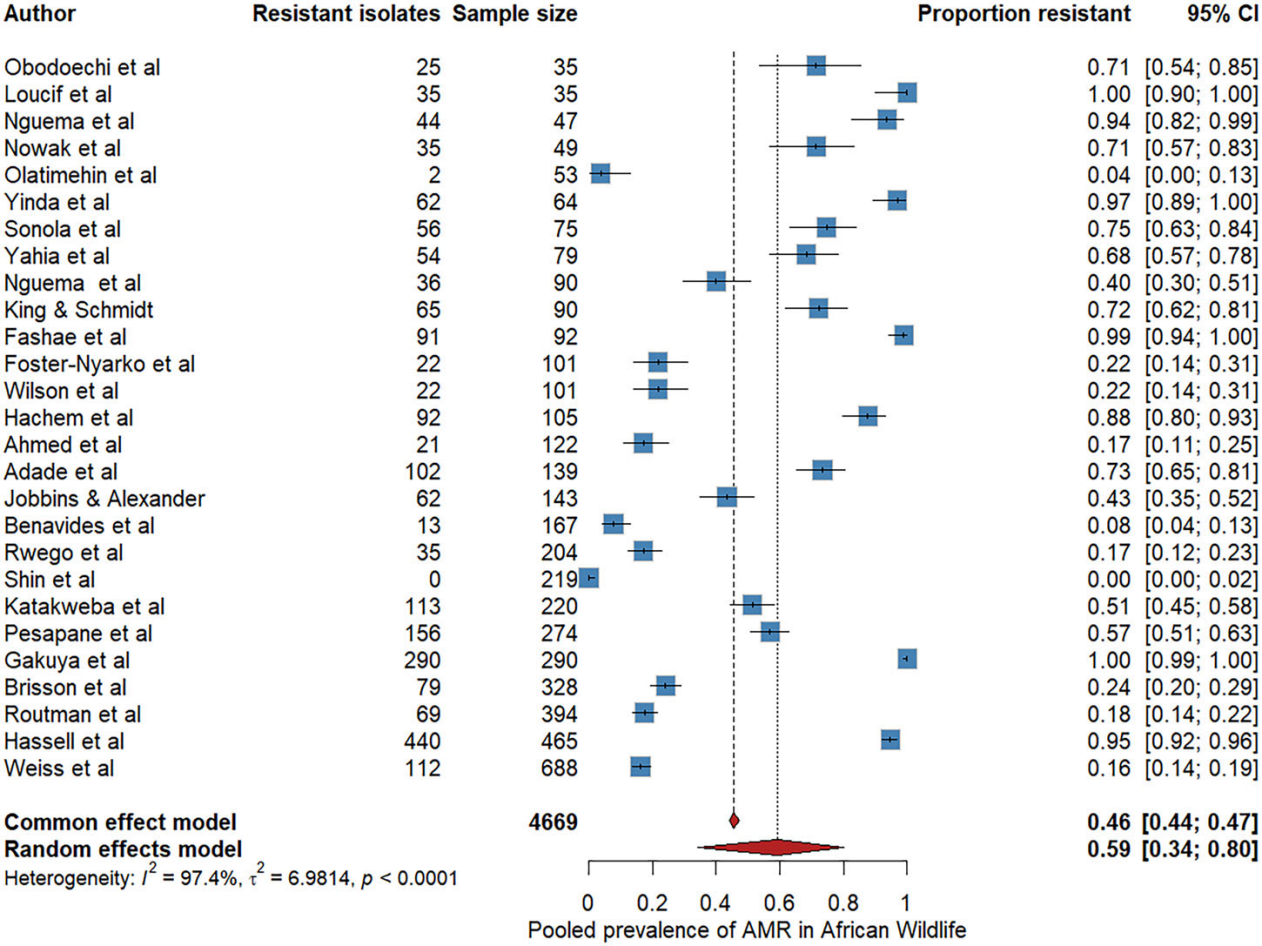
Forest plot showing the pooled prevalence of phenotypic antimicrobial resistance (AMR) in African wildlife with sample sizes ≥30 (*n* = 27). Blue squares represent study-specific prevalence estimates, with square size proportional to the study weight; horizontal whiskers denote 95% confidence intervals. The vertical dashed line marks the pooled estimate under the common-effects model, and the red diamond indicates the random-effects pooled prevalence (59%; 95% CI: 34–80%). High heterogeneity was observed (I² = 97.4%).

We performed a meta-analysis to estimate the pooled prevalence of AMR in bacterial isolates from African wildlife, restricted to studies with sample sizes ≥30 (*n* = 27). The meta-analysis included 4669 isolates, of which 2133 isolates were reported as resistant to at least one antimicrobial agent^12,13,21,26,27,30,32,33,35,41,43,45,46,48,49,52,57,61,63,65–68,70,73,76,78^. The random-effects model yielded a pooled prevalence of AMR of 59% (95% CI: 34–80%), with substantial heterogeneity across studies (I² = 97.4%). (Fig. 4). To account for temporal variability in diagnostic methods, antibiotic availability, and interpretive standards, we used 2010 as a pragmatic threshold, reflecting the period when Clinical and Laboratory Standards Institute (CLSI) and European Committee on Antimicrobial Susceptibility Testing (EUCAST) standards became more widely adopted, antimicrobial surveillance expanded, and antibiotic use practices began to shift. After excluding four studies conducted prior to 2010, the recalculated pooled prevalence was 65% (95% CI:38–85%; I^2^ = 96.6%).

To explore AMR prevalence variation by wildlife taxa, we stratified the analysis by the three most frequently reported host groups: herbivores, NHPs, and wild birds. Among herbivores (8 studies and 1218 isolates), the pooled AMR prevalence was 25% (95% CI: 9–54%, I² = 84.5%). For NHPs (10 studies and 2555 isolates), prevalence was higher at 35% (95% CI: 14**–**65.5%, I² = 97.4%). Wild birds (8 studies and 1330 isolates) showed the highest pooled prevalence, at 93% (95% CI: 70**–**99%, I² = 96.6%) (Supplementary Figs. 4–6).

### Pooled prevalence of *Escherichia coli* and multidrug resistance

**Fig. 5:**
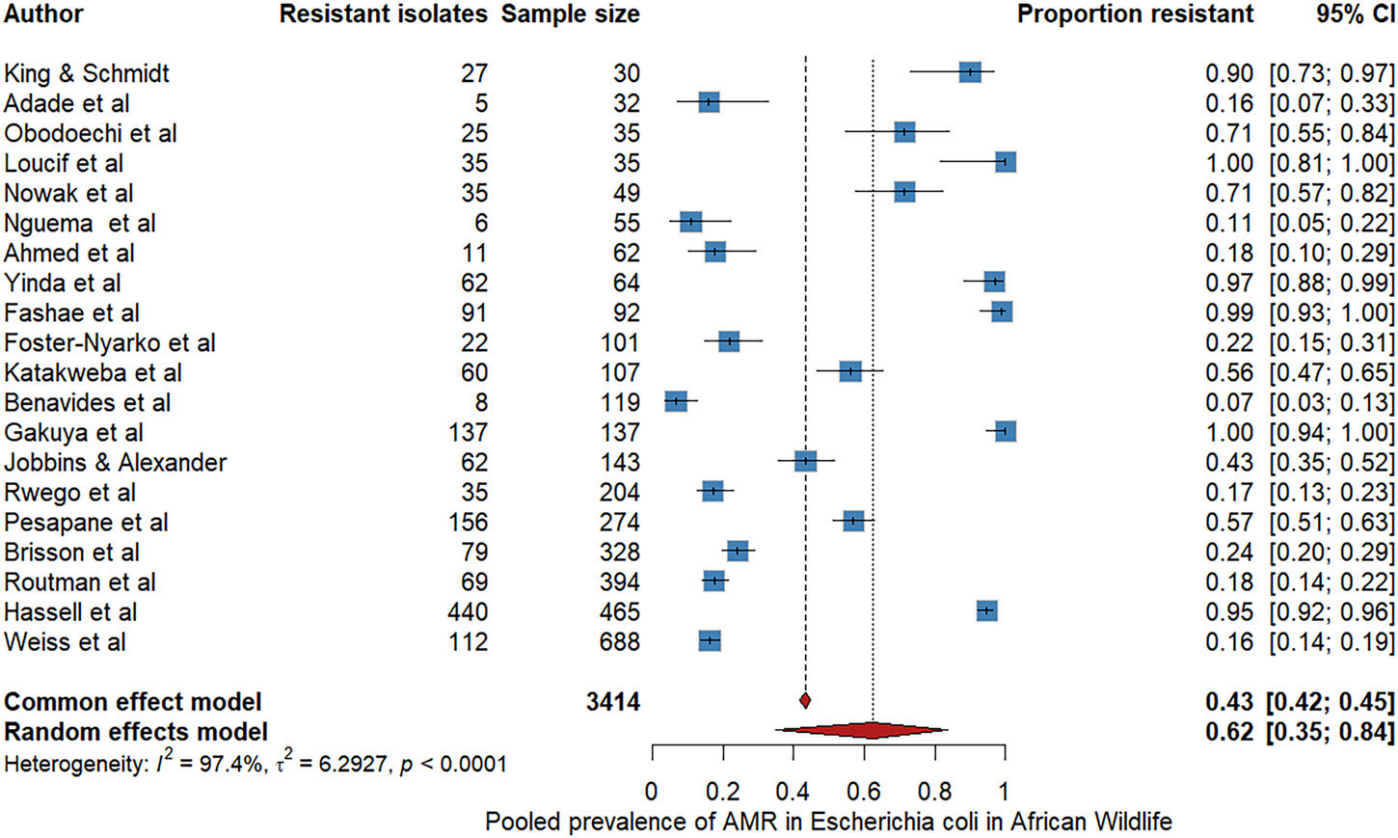
The forest plot displays data from 20 studies (n = 3414 isolates) reporting phenotypic AMR in *Escherichia coli* from diverse African wildlife species. Each blue square represents a study-specific prevalence estimate, with horizontal lines indicating 95% confidence intervals, while the red diamond denotes the pooled prevalence under a random-effects model. The pooled AMR in *E. coli* prevalence was 62% (95% CI: 35-84%, I^2^ = 97.2).

**Fig. 6:**
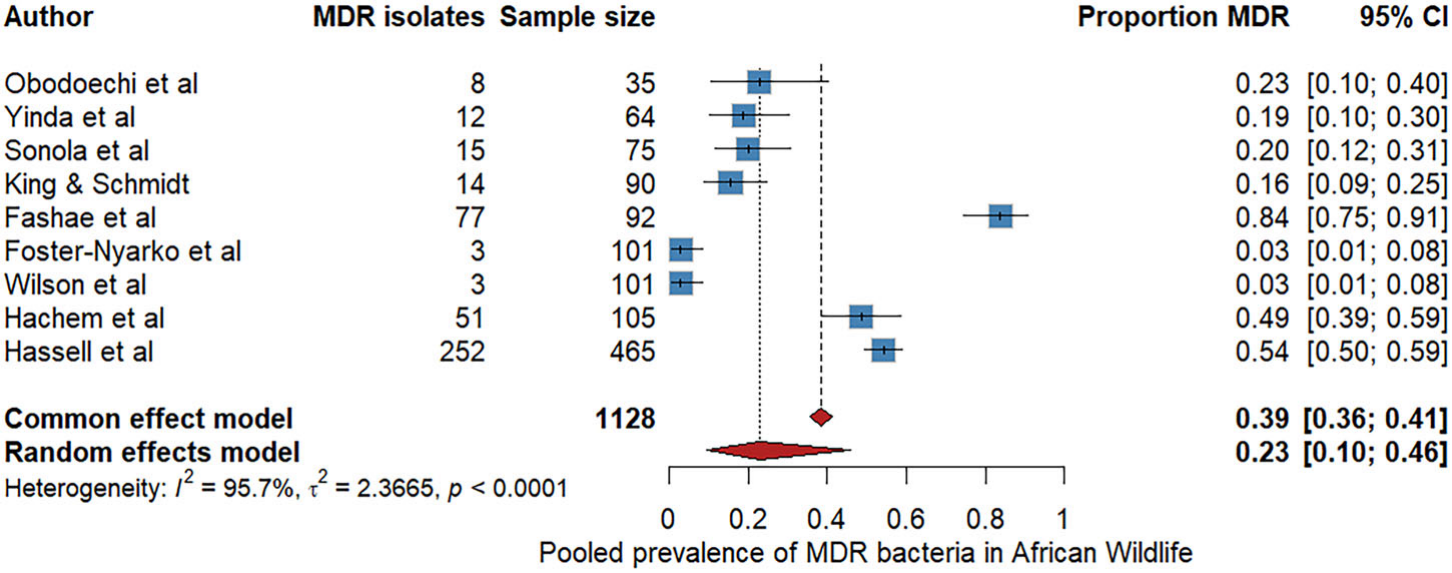
The forest plot presents nine studies (*n* = 1128 isolates) reporting multidrug-resistant (MDR) bacteria from wildlife in Africa. Each blue square represents an individual study estimate with 95% confidence intervals; the pooled prevalence derived under a random-effects model is shown as a red diamond. The pooled MDR prevalence was 23.1% (95% CI: 10–40%, I² = 95.7%).

We further performed bacteria-specific meta-analyses for *Escherichia coli*, the commonly studied bacterial species (Fig. 5). Twenty studies met the inclusion criteria, comprising 3414 isolates and 1468 resistance events^13,14,21,26,27,30,32,33,35,41,45,46,49,61,63,65,67,68,73,78^. The pooled prevalence under the random-effects model was 62% (95% CI: 35-84%, I^2^ = 97.2). Further, we conducted a separate meta-analysis focused on multidrug-resistant isolates (MDR) (Fig. 6). Definitions reported across included studies were consistent with the standard definition as previously described^80^, wherein MDR denotes non-susceptibility to at least one antimicrobial agent in three or more classes (Supplementary Table 6). This analysis included data from nine studies reporting MDR prevalence, comprising 438 MDR events among 1128 bacterial isolates^13,27,41,45,52,57,63,67,76^. The pooled MDR prevalence was 23.1% (95% CI: 10–40%, I² = 95.7%) using a random-effects model.

### Study quality and publication bias

**Fig. 7:**
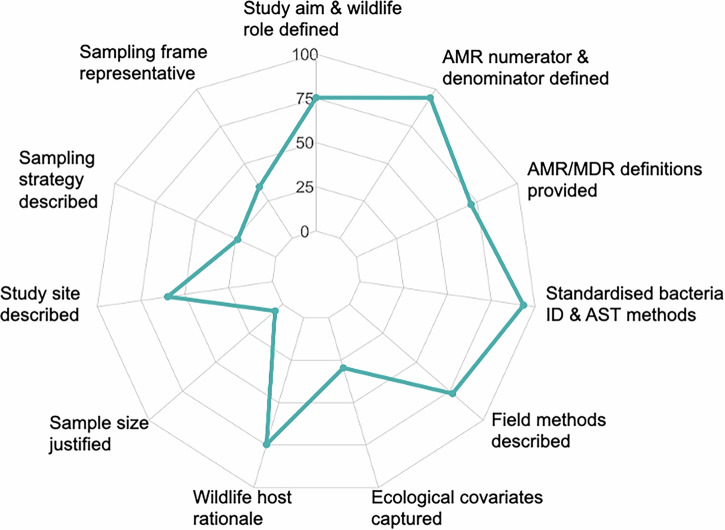
Radar plot of methodological quality across 11 appraisal items for the 61 wildlife AMR studies. Each axis shows the mean percentage of the maximum attainable score for that item (0–100%, where 1 = criterion fully met for all studies). Higher values indicate stronger reporting and lower risk of bias. Laboratory domains (standardised bacterial identification and AST methods, clear numerators/denominators, and AMR/MDR definitions) scored highest, whereas epidemiological and ecological domains (representative sampling frame, sampling strategy, ecological/behavioural covariates, and sample size justification) consistently scored lowest.

We evaluated all 61 included studies using an adapted 11-item quality appraisal checklist tailored for wildlife AMR surveillance (Fig. 7 and Supplementary Table 5). Overall, 47.5% (*n* = 29) of studies were categorised as high quality (score ≥7), 44.3% (*n* = 27) as moderate (score 4–7), and 8.2% (*n* = 5) as low quality (score <4). Methodological quality varied across the items. Most studies clearly stated their objectives (75.4%, *n* = 61), justified species selection (74.6%, *n* = 45.5), and described field collection procedures adequately (77%, *n* = 61). Laboratory performance was consistently strong: 93.4% (*n* = 57) of studies used standardised bacterial identification and antimicrobial susceptibility testing (AST) methods, and 94.3% (*n* = 57.5) clearly reported numerators, denominators, and resistance outcomes. By contrast, epidemiological and ecological domains showed substantial weaknesses. Only 34.4% (*n* = 21) of studies used a sampling frame that could reasonably be considered representative of the target wildlife population, and fewer than a quarter (23.8%, *n* = 14.5) reported an appropriate or justified sampling strategy. Ecological or behavioural covariates relevant to AMR exposure were captured in just 29.5% (*n* = 18) of studies. Formal sample size justification was present in (5.7%, *n* = 3.5) of studies.

To assess the influence of individual studies on the overall pooled prevalence, we performed a leave-one-out (LOO) sensitivity test (Supplementary Fig. 3). Sequential omission of each study produced minimal variation in the pooled prevalence of AMR which remained between 59 and 71% under the random-effects model (overall 65%; 95%CI: 38**–**85%, I^2^ = 96.6%). The values showed negligible change upon exclusion of any single study, indicating that no individual study disproportionately influenced the summary estimate.

## Discussion

We systematically reviewed the occurrence of AMR in wildlife hosts, analysing 61 studies from 21 African countries conducted between 1980 and 2025. The estimated prevalence of AMR varied, and meta-analysis revealed that 59% of bacterial isolates from diverse wild animal species were resistant to at least one antibiotic, and 23.1% exhibited multidrug resistance, indicating a widespread occurrence of AMR among wildlife populations. This relatively high prevalence, is broadly consistent with data from other global meta-analysis of wild animals, which reported a pooled prevalence of antibiotic resistance of 59.8%, and multidrug resistance prevalence of 17.2%^81^. Further, we observed differences in AMR prevalence across wildlife groups, reflective of variations in host natural history and ecological or behavioural drivers of AMR exposure^22,23^. Wild herbivores, which have relatively minimal exposure to anthropogenic activities, had the lowest pooled prevalence (25%)^82^, while non-human primates and wild birds exhibited higher prevalence (35 and 93%, respectively)^55^, likely reflecting greater ecological overlap with humans or higher mobility^23,83^. High heterogeneity (I² > 95%) observed across meta-analyses underscores the substantial methodological and ecological variability between studies, spanning geographic zones, host ecology, and AST standards, and cautions against over-interpreting pooled estimates. The presence of AMR bacteria in wildlife likely reflects exposure to anthropogenic and agricultural sources, mediated by behavioural and ecology traits such as animals’ feeding and foraging habits, social structure, and movement or migratory patterns; traits that can influence exposure to and acquisition of resistant bacteria and resistance genes from contaminated environments^84^. However, most studies did not collect or report these ecological and behavioural data, limiting the ability to interpret observed AMR patterns in relation to wildlife ecology or exposure pathways. The extent to which wild animals maintain, amplify, or disseminate acquired AMR bacteria and resistance determinants across One Health systems remains unclear, as does the risk of spillback from wildlife to humans or livestock^21^. Systematic risk assessment is urgently needed to accurately quantify the likelihood and magnitude of AMR transmission at the wildlife-human and wildlife-livestock-human interfaces. These assessments would help identify wildlife species or ecological contexts that pose the greatest transmission risks, if present, thus informing targeted surveillance and practical mitigation efforts.

A central conceptual debate within the AMR wildlife literature concerns whether wildlife should be viewed as vectors, victims, or sentinels of AMR (i.e. indicators of AMR contamination 'hotspots'). We propose a more nuanced approach: wildlife likely occupy overlapping and context-dependent roles in resistance dynamics, underscoring the need for a strategic reframing of wildlife AMR surveillance. Specifically, studies should articulate clear research objectives and explicitly address the rationale behind wildlife sampling. For example, is the primary aim to assess potential human health risks, evaluate threats to wildlife conservation, or use wildlife AMR prevalence as an indicator of broader environmental AMR contamination? Clearly defined objectives will, in turn, inform critical decisions such as the selection of target populations, ecological settings, microbial organisms of interest, appropriate laboratory methodologies, and necessary sample sizes. For instance, selecting wildlife species as AMR sentinels (i.e. to signal environmental contamination) should consider species-specific ecological traits such as habitat preference, foraging behaviour, home-range size, mobility patterns, proximity to anthropogenic interfaces, and ecological interactions with livestock or humans. The studies included in our review generally did not provide explicit justifications or discussions regarding their choice of wildlife taxa, highlighting an important gap and opportunity for improved study design in future research.

Current wildlife research is predominantly characterised by opportunistic, convenience-based sampling concentrated around conservation areas or human settlements, with only 21.1% of sampling sites in our review representing pristine or minimally disturbed habitats^16,22,84^. This potentially introduces significant bias when interpreting AMR patterns, as the data disproportionately reflect wildlife exposed to anthropogenic pressures. Additionally, sample sizes in our review varied considerably, ranging from as few as five animals^31^ to over 700^45^, often without adequate information regarding their representativeness, thus reducing both analytical power and the comparability of the findings. Therefore, efforts should be made to enhance the representativeness of collected samples and avoid convenience sampling without a clear study hypothesis. Instead, simple random sampling should be adopted wherever feasible, and studies should explicitly state how their findings can be generalised, clearly defining the target population and scope of inference. This approach is particularly critical given the inherent biological and ecological heterogeneity among wildlife species, habitats, and ecological contexts. Moreover, adequate statistical power is essential; thus, researchers should conduct appropriate sample size calculations that explicitly consider the population size of the target species and anticipated effect sizes. Such methodological rigour will facilitate accurate prevalence estimates and enhance the generalisability of the results^85^.

Pathogen selection in wildlife AMR research should be explicitly guided by clearly defined research objectives, whether the primary aim is to assess human health risks, threats to wildlife conservation, or environmental contamination levels. Our analysis revealed considerable bacterial diversity across studies, with *Escherichia coli* being the most frequently reported organism. Additionally, although 87% of included studies employed genotypic assays (predominantly PCR), few progressed to sequencing, limiting the resolution of resistance gene diversity. These findings reflect the predominance of anthropogenic indicator organisms commonly utilised in public health-oriented research. However, reliance on these anthropocentric indicators restricts our understanding of AMR ecology within wildlife populations and limits our ability to distinguish between transient colonisation and the maintenance of resistance within wildlife reservoirs. It also obscures the potential fitness costs and evolutionary implications of resistance within wildlife hosts. To address these gaps, future surveillance frameworks should broaden their scope to include wildlife-specific pathogens, particularly those pathogens known to cause clinical disease or impact wildlife health and conservation. This is essential to determine whether AMR is truly endemic within wildlife populations or merely a byproduct of environmental contamination.

Globally, research on AMR in wildlife remains limited, and wildlife is largely overlooked in AMR surveillance programmes and policy frameworks. This is particularly striking in Africa, the continent richest in wildlife diversity, home to more than 1100 mammal species (~17% of the world’s total) and over 2500 bird species^86^. Yet, despite its biodiversity, Africa remains underrepresented in the AMR wildlife literature, with only 61 studies meeting inclusion criteria over a 45-year period, originating from just 21 of the 54 African countries. This narrow geographic coverage highlights uneven research effort and the absence of systematic wildlife AMR surveillance in large parts of the continent. Future AMR surveillance should extend beyond treating wildlife as isolated entities; instead, wildlife components should be integrated into existing human clinical and agricultural surveillance systems wherever feasible. Only through such integrated surveillance can we effectively manage AMR; in line with the One Health approach, which emphasises leaving no sector behind. Most studies (88%) were published between 2012 and 2023, reflecting growing scientific interest; however, it remains unclear how AMR levels in wildlife are changing over time, and future work should include longitudinal studies that follow comparable populations using consistent methods to enable robust temporal assessment.

This review followed PRISMA guidelines and used comprehensive searches across four databases to generate an overview of AMR carriage in African wildlife. Several limitations, however, warrant consideration. The heavy reliance on convenience and faecal sampling, accounting for nearly 80% of studies, likely biased bacterial recovery toward readily culturable enteric organisms such as *Escherichia coli* and *Klebsiella pneumoniae*. Although operationally practical, this approach underrepresents organisms from other anatomical sites or environmental reservoirs and limits ecological interpretation. Considerable methodological heterogeneity was also evident across studies, including differences in antimicrobial susceptibility testing methods, interpretive standards (CLSI vs EUCAST), and antibiotic panels, reducing comparability and contributing to the high I² values observed in pooled analyses. Sampling designs varied widely, particularly across gradients of human and livestock exposure, further amplifying heterogeneity. Moreover, study distribution was geographically skewed, with northern and western Africa dominating the evidence base, whereas central, southern, and relatively undisturbed ecosystems remained poorly represented. Finally, restricting the search to English-language, peer-reviewed literature may have excluded relevant studies. These limitations highlight the need for harmonised, hypothesis-driven surveillance frameworks that use representative ecological sampling and standardised laboratory methods, and that prioritise underrepresented taxa and regions to improve understanding of AMR dynamics at the wildlife-livestock-human interface.

In conclusion, this systematic review demonstrates that AMR occurs across diverse African wildlife species, reflecting both environmental contamination and potential cross-species exchange. However, the predominant reliance on opportunistic and convenience-based sampling, conducted mainly within anthropogenically impacted settings and often lacking explicit rationale for target species or defined hypotheses, constrains ecological inference and limits understanding of baseline resistance in undisturbed systems. Wildlife should therefore not be viewed solely as vectors, victims, or sentinels of AMR, but rather as context-dependent participants within interconnected ecological networks. Advancing a One Health understanding of wildlife-associated AMR will require surveillance programmes guided by clearly defined research objectives: whether assessing human health risks, conserving wildlife populations, or monitoring environmental contamination. Such objectives will inform the choice of wildlife taxa, target organisms, and sampling designs. Furthermore, surveillance efforts must include currently underrepresented regions and pristine habitats, broaden focus to encompass wildlife-specific pathogens and diverse resistance determinants, and integrate longitudinal, genomic, and metagenomic approaches. Through standardised, hypothesis-driven, and ecosystem-oriented surveillance, it will be possible to more accurately characterise AMR reservoirs and elucidate transmission pathways involving wildlife. Expanding such efforts will be essential to characterise wildlife contributions to the broader AMR landscape and to inform evidence-based mitigation strategies across the human-animal-environment interface.

## Methods

### Search strategy and selection criteria

This systematic review was prospectively registered with the Open Science Framework (DOI: 10.17605/OSF.IO/KNC4R), no amendments were made to the protocol after registration^87^. The review was conducted and reported following the Preferred Reporting Items for Systematic Reviews and Meta-Analyses (PRISMA) guidelines^88^.

We systematically searched four electronic databases, MEDLINE/PubMed, Embase, BIOSIS and Web of Science, using combinations of Medical Subject Headings (MeSH) and free-text keywords related to AMR, wildlife hosts, and African countries. The initial search was performed on 6 August 2024 and updated on 16 April 2025. We restricted our searches to peer-reviewed studies published in English, with no limitations on publication date. Detailed search strategies for each database are provided in Supplementary Note 1.

To be eligible for inclusion, articles had to be original peer-reviewed research studies reporting primary data on AMR occurrence in wildlife hosts in Africa. We excluded, among others, reviews, prediction studies, and case reports. Likewise, studies on domesticated wild animals or animals farmed specifically for human consumption were excluded, as these are typically subject to direct human management and exposure to veterinary interventions, including routine antimicrobial use, which can substantially influence AMR profiles. Although systematic reviews and meta-analyses were excluded to avoid duplication of data, we manually screened their reference lists to identify additional relevant primary studies.

Two independent reviewers (JWM and DMM) screened the articles, initially by title and abstract, and subsequently by full-text assessment, to determine their final eligibility. Any disagreements during either screening phase were resolved through discussion and consensus. References identified through the searches were managed using Mendeley software, and duplicates were removed.

### Data extraction

Data extraction was conducted using a piloted, standardised template (Supplementary Table 3). Data were initially extracted from the full texts of eligible studies by one reviewer (JWM), with independent verification of all extracted data by a second reviewer (AK or DMM). Extracted variables included publication year, data collection date, country of sampling, study design, sample type, wildlife host species and sample sizes, bacterial species and isolate numbers, habitat type (wild or captive), antimicrobial susceptibility testing methods (phenotypic, genotypic, or both), and reported resistance profiles. The primary outcome of interest was the proportion of bacterial isolates reported as phenotypically resistant to at least one antibiotic or reported by the included studies as multidrug-resistant (MDR). For studies reporting MDR, we did not reclassify outcomes or redefine MDR categories. Instead, each study’s definition was reviewed and compared against the standard criterion as non-susceptibility to at least one agent in three or more antimicrobial categories^80^ (Supplementary Table 6). Wildlife hosts were defined as non-domesticated, free-living animal species not actively managed for human consumption or agricultural production. Wildlife taxa included in the analysis were grouped using a modified classification system based on functional groups or trophic levels, specifically bats, carnivores, non-human primates (NHPs), wild birds, rodents, herbivores, wild boars and pangolins.

### Risk of bias assessment

The methodological quality and risk of bias of the included studies were assessed using an adapted 11-item critical appraisal checklist derived from the Joanna Briggs Institute (JBI) critical appraisal checklists for cross-sectional and prevalence studies^89^.

The modified criteria incorporated both epidemiological and ecological dimensions relevant to wildlife AMR surveillance. Each study was assessed against eleven pre-defined domains, including the clarity of study objectives and the specified role of wildlife, representativeness of the sampling frame, appropriateness of the sampling strategy, adequacy of site description, justification of sample size, ecological and taxonomic justification for host selection, capture of behavioural and environmental covariates, rigour of field collection and contamination control, standardisation of bacterial identification and AST methods (e.g. CLSI/EUCAST), clarity of AMR or MDR definitions, and transparency in reporting numerators, denominators, and resistance outcomes (Supplementary Table 5).

Each criterion was scored as 1 (clearly met), 0.5 (partially met or unclear), or 0 (not met). The total quality score, therefore, ranged from 0 to 11, with higher scores indicating greater methodological rigour and lower risk of bias. Item-level scores for all eleven appraisal domains are presented in Supplementary Table 5, highlighting specific methodological strengths and potential sources of bias across studies.

Initial assessments were conducted independently by one reviewer (JM). A randomly selected 50% subset of studies was subsequently reassessed by a second reviewer (DMM) to ensure reliability. Discrepancies between reviewers were resolved through discussion and consensus. No studies were excluded based on the scores; however, the quality ratings were considered in the interpretation of meta-analytic results and subgroup analyses.

### Data analysis

A narrative synthesis was performed to summarise study characteristics, wildlife taxa, bacterial species, resistance profiles, and diagnostic methods. For quantitative synthesis, we conducted meta-analyses to calculate pooled prevalence estimates modelled via a logit transformation and inverse-variance weighting implemented in the *metaprop* function in the *meta* package in R (version 4.5.0). Studies were eligible for meta-analysis if they explicitly reported both the number of resistant isolates and the total number of bacterial isolates tested (*n* ≥ 30), thus enabling calculation of prevalence proportions. Only phenotypic resistance outcomes were included in meta-analyses. Subgroup meta-analyses were performed for *Escherichia coli*, the most frequently reported bacterial species. Additionally, we conducted a separate meta-analysis for multidrug-resistant isolates (as reported by the studies). Pooled prevalence estimates and their 95% confidence intervals were calculated using a random-effects model (between-study variance estimated using restricted maximum likelihood (REML)). Between-study heterogeneity was assessed using the I² statistic. Small-study effects and publication bias were visually assessed using funnel plots and statistically evaluated using Egger’s regression test. All results were visualised via forest and funnel plots (Supplementary Note 9).

## Supplementary information

Mwangi et al_Supplementary_File.

## Data Availability

All extracted data, summary tables, and analysis outputs generated during this study are included within the article and its Supplementary Information file. Additional materials are available from the corresponding author upon reasonable request.

## Code availability

No custom code was developed for this study. All analyses were conducted using standard functions in R (version 4.5.0), and analytical scripts are available upon reasonable request.
