# Supplementary material for: Prevalence and patterns of antimicrobial resistance among wildlife populations in Africa: a systematic review
Source: NPJ Antimicrob Resist. 2026 Feb 16;4:9. doi: 10.1038/s44259-025-00179-z (PMC12909803; doi:10.1038/s44259-025-00179-z)
Supplement: Supplementary file 1 — Mwangi et al_Supplementary_File. [file 44259_2025_179_MOESM1_ESM.docx]

**Supplementary File S1**

**Supplementary Material for *“Prevalence and patterns of antimicrobial resistance among wildlife populations in Africa: A systematic review”***

**Contents**

1. Supplementary note 1. Search Strategy
2. Supplementary note 2. PRISMA 2020 Checklist
3. Supplementary note 3. Summary of Included Studies
4. Supplementary note 4. Data Extraction Template
5. Supplementary note 5. Wildlife Host Functional Group Distribution
6. Supplementary note 5. Risk of Bias Assessment Summary
7. Supplementary note 6. Multidrug Resistance (MDR) Definitions and Supplementary note 7. Prevalence
8. Supplementary note 8. Reporting Limitations in Antibiotic Class–Specific Data
9. Supplementary note 9. Subgroup Meta-Analyses and Additional Figures
10. References

**Supplementary note 1. Search Strategy**

We conducted a comprehensive search in four electronic databases; MEDLINE/PubMed, Embase, BIOSIS, and Web of Science using a combination of Medical Subject Headings (MeSH) and free-text terms related to antimicrobial resistance (AMR), wildlife, and African countries. Searches were limited to English-language publications.

**Web of Science and Biosis search strings (executed 6th August 2024-** *The same search string was run in both Web of Science Core Collection and BIOSIS Citation Index***):**

(TS=AMR OR TS="Antibiotic Resistance" OR TS="Antimicrobial Resistance" OR TS="Drug resistance" OR TS=ABR) AND (TS=Algeria OR TS=Egypt OR TS=Libya OR TS=Morocco OR TS=Tunisia OR TS=Cameroon OR TS="Central African Republic" OR TS=Chad OR TS=Congo OR TS="Democratic Republic of Congo" OR TS="Equatorial Guinea" OR TS=Gabon OR TS=Sudan OR TS=Angola OR TS=Benin OR TS="Burkina Faso" OR TS="Cabo Verde" OR TS="Cote d'Ivoire" OR TS=Gambia OR TS=Ghana OR TS=Guinea OR TS="Guinea-Bissau" OR TS=Liberia OR TS=Mali OR TS=Mauritania OR TS=Niger OR TS=Nigeria OR TS=Senegal OR TS="Sierra Leone" OR TS=Togo OR TS=Kenya OR TS=Burundi OR TS=Comoros OR TS=Djibouti OR TS=Eritrea OR TS=Ethiopia OR TS=Madagascar OR TS=Malawi OR TS=Mauritius OR TS=Mozambique OR TS=Rwanda OR TS="Sao Tome and Principe" OR TS=Seychelles OR TS=Somalia OR TS="South Sudan" OR TS=Uganda OR TS=Tanzania OR TS=Zambia OR TS=Zimbabwe OR TS=Botswana OR TS=Eswatini OR TS=Swaziland OR TS=Lesotho OR TS=Namibia OR TS="South Africa") AND (TS=Lion OR TS=Giraffe OR TS="Wild Animal" OR TS=Wildlife OR TS=Monkey OR TS=Zebra OR TS=Elephant OR TS=Rhinoceros OR TS=Antelope OR TS="Wild Bird" OR TS=Vulture OR TS=Seagull OR TS=Rodent OR TS=Rat OR TS=Mice OR TS=Primate OR TS=Gorilla OR TS=Bat OR TS=Wildebeest OR TS=Buffalo OR TS=Mongoose)

**MEDLINE/PubMed search string**

((AMR or antibiotic resistance or antimicrobial resistance or drug resistance or ABR) and (algeria or egypt or libya or morocco or tunisia or cameroon or central african republic or chad or congo or democratic republic of congo or equatorial guinea or gabon or sudan or angola or benin or bukina faso or cabo verde or cote d'ivoire or gambia or ghana or guinea or guinea bissau or liberia or mali or mauritania or niger or nigeria or senegal or sierra leone or togo or kenya or burundi or comoros or djibouti or eritrea or ethiopia or madagascar or malawi or mauritius or mozambique or rwanda or seychelles or somalia or south sudan or uganda or tanzania or zambia or zimbabwe or botswana or eswatini or swaziland or lesotho or namibia or south Africa) and (lion or giraffe or wild animal or wildlife or monkey or zebra or elephant or rhinoceros or antelope or wild bird or vulture or seagull or rodent or rat or mice or primate or gorilla or bat or wildebeest or buffalo or mongoose)).mp. [mp=title, book title, abstract, original title, name of substance word, subject heading word, floating sub-heading word, keyword heading word, organism supplementary concept word, protocol supplementary concept word, rare disease supplementary concept word, unique identifier, synonyms, population supplementary concept word, anatomy supplementary concept word]

**EMBASE search string**

(TITLE-ABS-KEY(amr) OR TITLE-ABS-KEY(“antibiotic resistance”) OR TITLE-ABS-KEY(“antimicrobial resistance”) OR TITLE-ABS-KEY(“drug resistance”) OR TITLE-ABS-KEY(ABR)) AND (TITLE-ABS-KEY(Algeria) OR TITLE-ABS-KEY(Egypt) OR TITLE-ABS-KEY(Libya) OR TITLE-ABS-KEY(Morocco) OR TITLE-ABS-KEY(Tunisia) OR TITLE-ABS-KEY(Cameroon) OR TITLE-ABS-KEY(“central african republic”) OR TITLE-ABS-KEY(Chad) OR TITLE-ABS-KEY(Congo) OR TITLE-ABS-KEY(“democratic republic of congo”) OR TITLE-ABS-KEY(“equatorial guinea”) OR TITLE-ABS-KEY(Gabon) OR TITLE-ABS-KEY(Sudan) OR TITLE-ABS-KEY(Angola) OR TITLE-ABS-KEY(Benin) OR TITLE-ABS-KEY(“burkina faso”) OR TITLE-ABS-KEY(“cabo verde”) OR TITLE-ABS-KEY(“cote d'ivoire”) OR TITLE-ABS-KEY(Gambia) OR TITLE-ABS-KEY(Ghana) OR TITLE-ABS-KEY(Guinea) OR TITLE-ABS-KEY(“guinea bissau”) OR TITLE-ABS-KEY(Liberia) OR TITLE-ABS-KEY(Mali) OR TITLE-ABS-KEY(Mauritania) OR TITLE-ABS-KEY(Niger) OR TITLE-ABS-KEY(Nigeria) OR TITLE-ABS-KEY(Senegal) OR TITLE-ABS-KEY(“sierra leone”) OR TITLE-ABS-KEY(Togo) OR TITLE-ABS-KEY(Kenya) OR TITLE-ABS-KEY(Burundi) OR TITLE-ABS-KEY(Comoros) OR TITLE-ABS-KEY(Djibouti) OR TITLE-ABS-KEY(Eritrea) OR TITLE-ABS-KEY(Ethiopia) OR TITLE-ABS-KEY(Madagascar) OR TITLE-ABS-KEY(Malawi) OR TITLE-ABS-KEY(Mauritius) OR TITLE-ABS-KEY(Mozambique) OR TITLE-ABS-KEY(Rwanda) OR TITLE-ABS-KEY(“sao tome and principe”) OR TITLE-ABS-KEY(Seychelles) OR TITLE-ABS-KEY(Somalia) OR TITLE-ABS-KEY(“south sudan”) OR TITLE-ABS-KEY(Uganda) OR TITLE-ABS-KEY(Tanzania) OR TITLE-ABS-KEY(Zambia) OR TITLE-ABS-KEY(Zimbabwe) OR TITLE-ABS-KEY(Botswana) OR TITLE-ABS-KEY(Eswatini) OR TITLE-ABS-KEY(Swaziland) OR TITLE-ABS-KEY(Lesotho) OR TITLE-ABS-KEY(Namibia) OR TITLE-ABS-KEY(“south africa”)) AND (TITLE-ABS-KEY(Lion) OR TITLE-ABS-KEY(Giraffe) OR TITLE-ABS-KEY(“wild animal”) OR TITLE-ABS-KEY(Wildlife) OR TITLE-ABS-KEY(Monkey) OR TITLE-ABS-KEY(Zebra) OR TITLE-ABS-KEY(Elephant) OR TITLE-ABS-KEY(Rhinoceros) OR TITLE-ABS-KEY(Antelope) OR TITLE-ABS-KEY(“wild bird”) OR TITLE-ABS-KEY(Vulture) OR TITLE-ABS-KEY(Seagull) OR TITLE-ABS-KEY(Rodent) OR TITLE-ABS-KEY(Rat) OR TITLE-ABS-KEY(Mice) OR TITLE-ABS-KEY(Primate) OR TITLE-ABS-KEY(Gorilla) OR TITLE-ABS-KEY(Bat) OR TITLE-ABS-KEY(Wildebeest) OR TITLE-ABS-KEY(Buffalo) OR TITLE-ABS-KEY(Mongoose))

An updated search across al databases was conducted in April 2025 to include the most recent literature.

**Supplementary note 2. PRISMA 2020 Checklist**

All relevant PRISMA 2020 checklist items were addressed in the main manuscript to ensure transparency and adherence to established systematic review reporting standards. The following checklist summarises the key reporting items from the PRISMA 2020 guidelines. Each item is linked to the corresponding section of the main manuscript.

Supplementary Table 1. PRISMA 2020 Checklist for Systematic Reviews.
This table outlines the required reporting elements based on the PRISMA 2020 guidelines, mapped to their respective locations in the manuscript to ensure comprehensive and transparent reporting.

| Section and Topic | Checklist Item | Page Number in Manuscript |
| --- | --- | --- |
| Title | Identify the report as a systematic review. | 1 |
| Abstract | Provide a structured summary including background, objectives, data sources, eligibility criteria, methods, results, limitations, conclusions, and registration number. | 2 |
| Introduction | Describe the rationale for the review.  Provide an explicit statement of the objectives or questions addressed. | 2-3 |
| Methods | Specify the inclusion and exclusion criteria. | 3-4 |
| Methods | Describe all information sources (databases, registers, etc.) and the date of the last search. | 4 |
| Methods | Present the full search strategies for all databases and sources. | 4 |
| Methods | Specify the methods used to select studies. | 4 |
| Methods | Specify the data collection process. | 4-5 |
| Methods | List and define all variables for which data were sought. | 5 |
| Methods | Describe methods used to assess risk of bias in included studies. | 6 |
| Methods | Describe methods of synthesis. | 6-7 |
| Results | Provide a summary of included studies. | 8 |
| Results | Present results of individual studies and any synthesis. | 8 |
| Results | Present results of any additional analyses (e.g., subgroups, sensitivity). | 8 -15 |
| Results | Present results of risk of bias assessment. | 15-16 |
| Discussion | Summarize main findings including strength of evidence. | 17-19 |
| Discussion | Discuss limitations of the evidence and the review process. | 20 |
| Discussion | Provide implications for practice, policy, and future research. | 20-21 |
| Other | Provide registration information and protocol access (if available). | 4 |
| Other | Describe sources of funding and role of funders. | 21 |
| Other | Disclose conflicts of interest. | 21 |

**Supplementary note 3. Summary of Included Studies**

Supplementary Table 2. Summary of Included Studies Reporting Antimicrobial Resistance in African Wildlife
This table provides an overview of the 61 studies included in the systematic review, capturing study-level variables such as country, bacterial species, host species, sample type, habitat context, sample size, AMR outcomes, sampling strategy, phenotypic methods used, and interpretive criteria.

| # | Study | Country | Resistant bacteria reported | Wild animals studied | Reclassified functional groups | Sample type | Site | Animal sample size | AMR reported | MDR reported | Sampling strategy | Phenotypic assay used | Standard interpretative criteria used |
| --- | --- | --- | --- | --- | --- | --- | --- | --- | --- | --- | --- | --- | --- |
| 1 | 1- Bachiri et al^1^ | Algeria | *Escherichia coli* | Barbary Macaques | NHP | Faecal | Community area | 86 | 100% |  | Active | Disk diffusion | EUCAST |
| 2 | Bachiri et al^2^ | Algeria | *Escherichia coli, Klebsiella pneumoniae* | Wild Boars & Barbary Macaques | Wild boars; NHP | Faecal | Wild | 380 | 100% |  | Mixed (active and passive) | Disk diffusion | EUCAST |
| 3 | Bellil et al^3^ | Algeria | *Salmonella bredeney, Salmonella enteritidis, Salmonella altona, Salmonella virchow* | Barbary Monkeys, Wild Boars, Barbary Deer, Wild Birds | NHP; Wild boars; Herbivores; Wild birds | Faecal | Conservation area | 426 | 50% |  | Active | Disk diffusion | CLSI |
| 4 | Gharout-Sait et al^4^ | Algeria | *Klebsiella pneumoniae* | Bats | Bats | Faecal | Conservation area | 110 | 100% |  | Active | Disk diffusion | EUCAST |
| 5 | Hachem et al^5^ | Algeria | *Enterococcus casseliflavus, Enterococcus gallinarum, Enterococcus faecalis* | Captive, Semi Captive, Wild Living Birds | Wild birds | Cloacal and faecal samples | Community area | 444 | 88% | 55% | Active | Disk diffusion | CLSI /EUCAST (imipenem) |
| 6 | Loucif et al^6^ | Algeria | *Escherichia coli* | Pigeon | Wild birds | Faecal | Wild | 276 | 100% |  | Active | Disk diffusion | CLSI &EUCAST (Colistin) |
| 7 | Mairi et al^7^ | Algeria | *Staphylococcus aureus* | Bats | Bats | Faecal | Community area | 98 | 36% | 36% | Active | Disk diffusion | EUCAST |
| 8 | Jobbins & Alexander^8^ | Botswana | *Escherichia coli* | Crocodile, Spotted Hyena, Leopard, Otter, Cape Buffalo, Bushbuck, Elephant, Giraffe, Hippopotamus, Impala, Greater Kudu, Sable, Waterbuck, Chacma Baboon, Guineafowl, Banded Mongoose, Vervet Monkey and Warthog | Reptiles; Carnivores; Herbivores; NHP; Wild birds | Faecal | Conservation area | 143 | 43% | 14% | Active | Disk diffusion | CLSI |
| 9 | Pesapane et al^9^ | Botswana | *Escherichia coli* | Banded Mongoose | Carnivores | Faecal | Community area | 179 | 57% |  | Active | Disk diffusion | CLSI |
| 10 | Janatova et al^10^ | Central African Republic | *Escherichia coli, Klebsiella pneumoniae* | Western lowland gorillas, habituated agile mangabeys, unhabituated central chimpanzees, African buffalos, forest elephants, red river hogs, Peter’s duikers, lowland bongos, sitatunga, blue duikers | NHP; Herbivores | Faecal | Conservation area | 89 |  |  | Active | Disk diffusion | Not specified |
| 11 | Albrechtova et al^11^ | Coate d'Ivoire | *Citrobacter freundii, Klebsiella pneumoniae, Enterobacter asburiae* | Non-human Primates, Mice | NHP; Rodents | Faecal | Community area | 79 | 0% |  | Active | Disk diffusion | CLSI |
| 12 | Klee et al^12^ | Coate d'Ivoire, Cameroon | *Bacillus anthracis* | Chimpanzees, Gorilla | NHP | Organ samples (e.g., spleen, lung, liver, bone marrow, tooth) | Wild | 5 | 100% |  | Passive | Disk diffusion, capsule formation, gamma phage sensitivity | Not specified |
| 13 | Nowak et al^13^ | Congo | *Escherichia coli* | Fruit Bats | Bats | Tissue samples (liver, lung and intestines | Community area | 50 | 71% |  | Active | Disk diffusion | CLSI |
| 14 | Ahmed et al^14^ | Egypt | *Escherichia coli, Klebsiella pneumoniae, Klebsiella oxytoca, Pseudomonas aeruginosa* | Wild Birds (Hooded Crows, Cattle Egrets, Rock Pigeons, Laughing Dove, Migratory Waterfowls (Shoveler Ducks, Cotte Ducks and Green-Winged Teal Ducks) | Wild birds | Faecal | Community area & Wild | 140 | 17% |  | Active | Disk diffusion | Not specified |
| 15 | Eidaroos et al^15^ | Egypt | *Staphylococcus aureus* | Rodents | Rodents | Nasal and mouth swabs, heart blood, intestinal content and lung tissues | Community area | 56 | 100% |  | Active | Disk diffusion | CLSI |
| 16 | Fadel et al^16^ | Egypt | *Shiga toxin Producing Escherichia Coli* | Pigeons, House Crows, Cattle Egrets, Moorhens, and House Teals | Wild birds | Intestinal content swabs | Wild & Conservation area | 177 | 100% |  | Active | Disk diffusion | Not specified |
| 17 | Raheem et al^17^ | Egypt | *Vibrio parahaemolyticus* | Migratory Birds | Wild birds | Cloacal swab | Community area | 80 | 18% | 27% | Active | Disk diffusion | Not specified |
| 18 | 1- Nguema et al^18^ | Gabon | *Achromobacter xylosoxidans, Providencia* | Wild Western Lowland Gorillas | NHP | Fecal | Community area | 27 | 100% |  | Active | Broth Microdilution / MIC | CLSI |
| 19 | Nguema et al^19^ | Gabon | *Escherichia coli* | Western lowland gorillas, chimpanzees, mandrills, monkeys (black colobus and gray-cheeked mangabey), duikers of several species, river hogs, forest buffalos, African elephants | NHP; Herbivores | Faecal | Conservation area | 125 | 40% |  | Active | Agar Disk diffusion | EUCAST |
| 20 | Nguema et al^20^ | Gabon | *Enterobacter cloacae, Proteus mirabilis, Klebsiella variicola, Klebsiella aerogenes, Klebsiella oxytoca, Citrobacter freundii, Klebsiella pneumoniae, Serratia marcescens* | Western lowland gorillas, mandrills, red-capped mangabeys, greater spot-nosed monkeys, black colobus monkeys, various species of duikers, common genets, waterbucks, African forest elephants, African buffalos, and red river hogs | NHP; Herbivores | Faecal | Wild | 68 | 100% |  | Active | Disk diffusion | EUCAST |
| 21 | Nguema et al^21^ | Gabon | *Escherichia coli, Klebsiella pneumoniae, Enterobacter cloacae, Enterobacter aerogenes, Serratia plymuthica, Citrobacter freundii, Enterobacter hormaechei, Ewingella americana, Morganella morganii, Pantoea, Proteus vulgaris* | Fruit Bats | Bats | Faecal | Community area | 277 | 94% |  | Active | N/A | CLSI |
| 22 | Wiethoff et al^22^ | Gabon | *Cedecea davisae, Enterobacter cancerogenus, Enterobacter cloacae, Enterobacter ludwigii, Klebsiella pneumoniae, Klebsiella variicola, Escherichia coli* | Lowland Gorrilas | NHP | Pharyngeal Swab | Conservation area | 89 | 0% |  | Mixed active and passive | Automated system (VITEK), anti-microbial susceptibility testing (AST) test cards, Disk diffusion | EUCAST |
| 23 | Yinda et al^23^ | Gabon | *Escherichia coli, Klebsiella pneumoniae, Achromobacter, Pseudomonas aeruginosa, Pseudomonas putida, Stenotrophomonas maltophilia, Stenotrophomonas, Pseudomonas* | Pangolin | Pangolin | Faecal | Wild | 96 | 97% | 19% | Active | Disk diffusion | CLSI |
| 24 | Benavides et al^24^ | Gabon | *Escherichia coli* | Western Lowland Gorillas (Gorilla Gorilla Gorilla) | NHP | Faecal | Conservation area | 167 | 8% |  | Active | Disk diffusion | CLSI |
| 25 | Foster-Nyarko et al^25^ | Gambia | *Escherichia coli* | Guinea baboons, green monkeys, red colobus monkeys, and patas monkeys | NHP | Faecal | Wild & Community | 43 | 22% | 3% | Active | Agar dilution | EUCAST |
| 26 | Adade et al^26^ | Ghana | *Staphylococcus haemolyticus, Staphylococcus gallinarum, Staphylococcus sciuri, Escherichia coli, Klebsiella pneumoniae Ssp. Pneumoniae* | Non-Human Primates | NHP | Oral and Rectal swabs | Community area | 85 | 73% |  | Active | Disk diffusion | Not specified |
| 27 | Modupe et al^27^ | Ghana | *Shigella, Yersinia, Salmonella, Citrobacter, Enterobacter, Klebsiella, Proteus, Pseudomonas, Serratia, Escherichia* | Wild Birds | Wild birds | Cloacal Swab | Conservation area | 138 | 100% | 0% | Active | Disk diffusion | CLSI |
| 28 | Schaufler et al^28^ | Guinea | *Esbl klebsiella Pneumoniae, Esbl escherichia Coli* | Rats | Rodents | Rectal swabs | Community area | 29 | 100% |  | Active | Disk diffusion | CLSI |
| 29 | Gakuya et al^29^ | Kenya | *Escherichia coli, Salmonella typhimurium, Klebsiella pneumoniae, Enterobacter cloacae, Enterobacter sakazakii, Citrobacter freundii, Morganella morganii, Pseudomonas aeruginosa, Burkholderia cepacia* | Rats | Rodents | Necropsied (Liver, spleen & Intestines) | Community area | 215 | 100% |  | Active | Disk diffusion | Not specified |
| 30 | Hassell et al^30^ | Kenya | *Escherichia coli* | Bats, Birds, Nhp And Rodents | Bats; Wild birds; NHP; Rodents | Faecal | Community area | 767 | 95% |  | Active | Disk diffusion | CLSI |
| 31 | Kipkorir et al^31^ | Kenya | *Escherichia coli* | Black Rhinoceros | Herbivores | Faecal | Wild, Conservation area, and Community area | 16 | 100% |  | Active | Disk diffusion | CLSI |
| 32 | Clausen & Ashford^32^ | Kenya & Tanzania | *Streptococcus, Staphylococcus aureus, Salmonella, Escherichia coli, Klebsiella, Aeromonas, Pseudomonas, Proteus* | Black Rhinoceros | Herbivores | Skin swab, saliva, genitalia swab | Community area | 30 | 100% |  | Active | Not Specified | Cowan & Steel & Breed et al., |
| 33 | Qiao et al^33^ | Madagascar | *Lactiplantibacillus plantarum* | Lemur | NHP | Faecal | Community area | 17 | 100% |  | Active | Disk diffusion | EUCAST, EFSA for Lp. Plantarum |
| 34 | Adesoji et al^34^ | Nigeria | *Mammaliicoccus sciuri, Staphylococcus gallinarum, Staphylococcus nepalensis* | Bats | Bats | Pharyngeal Swab | Wild | 23 | 100% |  | Active | Disk diffusion | Not Specified |
| 35 | Akobi et al^35^ | Nigeria | *Staphylococcus aureus* | Bats | Bats | Faecal | Conservation area | 560 |  |  | Active | Disk diffusion | CLSI |
| 36 | Eze et al^36^ | Nigeria | *Citrobacter freundii, Klebsiella pneumoniae* | Rats, grasscutters, squirrel, antelopes, rabbits and Lizard | Rodents; Herbivores; Wild rabbits; Reptiles | Necropsied | Conservation area | 42 |  |  | Active | Disk diffusion | Not specified |
| 37 | Fashae et al^37^ | Nigeria | *Escherichia coli* | Wild Birds (Cattle Egrets, Bubulcus Ibis, White-Faced Whistling Duck) | Wild birds | Faecal | Community area | 52 | 99% | 84% | Active | Disk diffusion | CLSI |
| 38 | Obodoechi et al^38^ | Nigeria | *Escherichia coli* | Bats | Bats | Liver, spleen, intestines | Community area | 180 | 71% | 23% | Active | Disk diffusion, DDST | CLSI |
| 39 | Okunlade^39^ | Nigeria | *Escherichia coli* | Green Monkeys, Patas Monkey, White Throated Monkey, Mona Monkey And Anubis Baboon | NHP | Faecal | Wild | 43 | 100% |  | Active | Automated system (VITEK) | CLSI |
| 40 | Olatimehin et al^40^ | Nigeria | *Staphylococcus aureus Complex, Staphylococcus aureus, Staphylococcus schweitzeri, Staphylococcus argenteus* | Fruit Bats | Bats | Faecal | Community area | 250 | 4% |  | Passive | Automated system (VITEK) | EUCAST |
| 41 | Abdallah et al^41^ | Senegal | *Pediococcus pentaceus, Weissela paramesenteroides, Lactobacillus plantarum, Lactobacillus brevis, Bacillus magaterium, Enterococcus gallinarum, Enterococcus hirae, Enterococcus avium, Enterococcus faecium, Pseudomonas monteilii, Pseudomonas guariconensis, Pseudomonas citronellolis, Pseudomonas aeruginosa, Pseudomonas nitroreducens, Pseudomonas putida, Pseudomonas oryzihabitans, Stenotrophomonas maltophilia, Acinetobacter courvalini, Acinetobacter bereziniae, Acinetobacter guillouie, Acinetobacter pittii, Acinetobacter gyllenbergi, Acinetobacter tandoii, Acinetobacter johsonii, Acinetobacter nosocomialis, Acinetobacter ursingii, Acinetobacter gerneri, Acinetobacter otitidis, Acinetobacter baumannii, Brucella intermedium, Bordetella trematum, Castenellaniella defragans, Achromobacter insolitus, Achromobacter xylosoxidans, Enterobacterales, Serratia marcescens, Escherichia coli, Enterobacter sp., Morganella morganii, Proteus mirabilis, Providencia alcalifaciens, Providencia rettgeri* | Monkeys, Baboon, Chimpanzees | NHP | Faecal | Community area | 226 |  |  | Active | Disk diffusion, E-test, UMIC microdilution | EUCAST |
| 42 | Glover et al^42^ | South Africa | *Escherichia fergusonii* | Non-Human Primates (Vervets And Baboons) | NHP | Faecal | Community area | 300 | 65% | 12% | Active | Broth Microdilution / MIC | EUCAST |
| 43 | King & Schmidt^43^ | South Africa | *Escherichia coli* | Giraffe, Zebra and Wildebeest | Herbivores | Faecal | Conservation area | 150 | 72% | 47% | Active | Disk diffusion | EUCAST |
| 44 | More et al^44^ | South Africa | *Campylobacter jejuni, Campylobacter lari, Salmonella enterica subsp enterica* | Kelp Gulls and greater crested terns | Wild birds | Faecal | Conservation area | 229 |  |  | Active | Disk diffusion | CLSI |
| 45 | Wilson et al^45^ | South Africa | *Staphylococcus aureus* | Pigeon | Wild birds | Faecal & rectal swabs | Community area | 43 | 22% | 3% | Active | Disk diffusion | CLSI |
| 46 | Katakweba et al^46^ | Tanzania | *Escherichia coli, Enterococcus faecium* | Buffalo, Wildebeest, Zebra | Herbivores | Faecal | Conservation area | 115 | 51% |  | Active | Disk diffusion | CLSI |
| 47 | Routman et al^47^ | Tanzania | *Escherichia coli* | Free-Ranging African Yellow Baboons | NHP | Faecal | Community area | 394 | 18% |  | Active | Replica plating | Not mentioned |
| 48 | Shin et al^48^ | Tanzania | *Enterococcus avium, Enterococcus casseliflavus, Enterococcus faecalis, Enterococcus faecium, Enterococcus hirae, Enterococcus mundtii* | African Buffalo | Herbivores | Ear and rectal swabs | Community area | 209 | 0% |  | Passive | Broth Microdilution / MIC | CLSI |
| 49 | Sonola et al^49^ | Tanzania | *Staphylococcus aureus* | Rodents | Rodents | Deep pharyngeal swabs & intestinal contents | Community area | 101 | 75% | 27% | Active | Disk diffusion | CLSI |
| 50 | Klibi et al^50^ | Tunisia | *Enterococcus faecium, Enterococcus faecalis, Enterococcus casseliflavus, Enterococcus gallinarum, Enterococcus durans* | Wild birds | Wild birds | Faecal (Clocal swabs) | Conservation area | 111 |  |  | Active | Disk diffusion | CLSI |
| 51 | Said et al^51^ | Tunisia | *Escherichia coli, Enterococcus hirae, Enterococcus faecalis, Enterococcus faecium* | Wild Rabbits | Wild rabbits | Rectal swabs | National Park | 49 | 0% |  | Active | Disk diffusion | Not specified |
| 52 | Selmi et al^52^ | Tunisia | *Escherichia coli, Citrobacter freundii, Klebsiella oxytoca, Klebsiella pneumoniae* | Wild Boars | Wild boars | Faecal | Wild | 110 |  | 56% | Active | Disk diffusion | EUCAST |
| 53 | Tayh et al^53^ | Tunisia | *Shiga toxin producing Escherichia coli (STEC)* | Wild boars | Wild boars | Faecal | Conservation area | 110 |  | 42% | Active | Disk diffusion | Not specified |
| 54 | Yahia et al^54^ | Tunisia | *Escherichia coli* | Wild Birds | Wild birds | Faecal | Community area | 111 | 100% |  | Active | Disk diffusion | CLSI |
| 55 | Yahia et al^55^ | Tunisia | *Enterococcus faecalis, Enterococcus faecium, Enterococcus casseliflavus* | Wild Birds | Wild birds | Faecal & pellet rejection | Conservation area | 150 | 68% |  | Active | Disk diffusion | CLSI |
| 56 | Rwego et al^56^ | Uganda | *Escherichia coli* | Mountain Gorillas | NHP | Faecal | Wild | 66 | 17% |  | Active | Disk diffusion | CLSI |
| 57 | Weiss et al^57^ | *Uganda* | *Escherichia coli* | Mountain Gorilla, Black-And-White Colobus, Red Colobus, Red-Tailed Guenon, Chimpanzee | NHP | Faecal | Conservation area |  | 16% |  | Active | Disk diffusion | CLSI |
| 58 | Kabali et al^58^ | Zambia | *Escherichia coli* | Blue wildebeest, buffalo, bushbuck, eland, hartebeest, impala, Kafue lechwe, puku, roan antelope, sable antelope, ostriches, and the common tsessebe | Herbivores; Wild birds | Faecal | Community area | 36 | 34% |  | Active | Disk diffusion | CLSI |
| 59 | Mubita et al^59^ | Zambia | *Salmonella garoli, Salmonella pomona, Salmonella roan* | Leopard, sable, Impala | Carnivores; Herbivores | Faecal | Community area | 240 |  |  | Mixed (active and passive) | Disk diffusion | CLSI |
| 60 | Schaumburg et al^60^ | Zambia & Uganda | *Staphylococcus aureus* | Apes | NHP | Oral and nasal swabs | Wild | 62 |  |  | Active | Automated system (VITEK) | Not specified |
| 61 | Brisson et al^61^ | Zimbabwe | *Escherichia coli* | Impala, Greater Kudu, and Plains Zebra | Herbivores | Faecal | Community area | 137 | 24% | 9% | Active | Disk diffusion | CLSI |

**Supplementary note 4. Data extraction template**

This data extraction template was developed to systematically capture study-level and outcome-level variables relevant to antimicrobial resistance (AMR) in African wildlife populations. Variables were selected to reflect methodological quality, host ecology, bacterial taxa, and antimicrobial susceptibility testing (AST) practices, as well as to support subgroup and quantitative synthesis. Each entry was derived from full-text review of eligible studies and cross-verified for internal consistency. Standardised coding (e.g., for host categories, AST methods, and sample type) was applied to facilitate harmonised analysis across diverse study designs.

Supplementary Table 3 Structured data extraction template for studies reporting antimicrobial resistance (AMR) in African wildlife. Each variable is defined and accompanied by a representative example to guide consistent data entry across included studies. This framework underpinned the descriptive synthesis and meta-analysis presented in the main review.

| Variable | Description | Example Entry |
| --- | --- | --- |
| Study ID | Sequential numbering as unique identifier | 2024-009 |
| First Author | Unique identifier for quantitative analysis (e.g., Author et al) | Gakuya et al |
| DOI or Reference | Data Source | 10.4314/eamj.v78i12.8934 |
| Country | Country where the study was conducted | Kenya |
| Study setting | Habitat type (e.g., community area, conservation area, wild) | Community area |
| Host species | Common name | Vervet monkey |
| Functional group (host category) | Taxonomic grouping used in analysis (e.g., herbivore, wild bird, NHP) | Non-human primate |
| Study Design | Employed study design | Cross-sectional |
| Sample size (animals) | Number of animals sampled | 215 |
| Bacterial species | Species of bacteria isolated and tested | Escherichia coli |
| Number of bacterial isolates | Total isolates included in AST | 215 |
| Spatio-temporal correlation | Correlation or pattern reported yes/no | No |
| Health status | Condition of the animals sampled | Healthy |
| Pooled or individual | Samples tested as individual or pooled | pooled |
| Phenotypic testing | Yes/no | Yes |
| Phenotypic AST method | Method used for susceptibility testing | Disk diffusion |
| Genotypic method used | PCR, WGS, not used, etc. | PCR |
| AST interpretation standard | CLSI, EUCAST, others or not specified | CLSI |
| Antibiotics tested | Antibiotics included in testing | Ampicillin, Gentamicin, Ciprofloxacin |
| AMR prevalence (%) | Reported AMR prevalence | 84% |
| MDR prevalence (%) | Reported MDR prevalence as defined by the study | 14% |
| Sampling strategy | Active or passive | Active |
| Additional notes | Any other observations (e.g., missing data, multiple hosts) | Mixed wildlife species |

**Supplementary note 5. Wildlife Host Functional Group Distribution**

To characterise the taxonomic breadth of wildlife represented in the included studies, all host species were reclassified into functional groups based on shared ecological or trophic attributes. This allowed harmonised comparison across studies that sampled multiple species or reported heterogeneous taxa. Supplementary Table 4 summarises the number and proportion of studies that included each functional group.

Supplementary Table 4. Distribution of the 61 studies included in the systematic review across wildlife host functional groups. Values represent the number of studies that sampled at least one species within each functional group. Percentages indicate the proportion of included studies investigating each host group*. Individual studies may be represented in multiple categories when more than one wildlife group was sampled. *Percentages calculated as n / 61 × 100, rounded to one decimal place.

| Wildlife host functional group | Number of studies (n) | Proportion of included studies (%*) |
| --- | --- | --- |
| Non-human primates (NHP) | 23 | 37.7 |
| Wild birds | 16 | 26.2 |
| Herbivores | 13 | 21.3 |
| Bats | 9 | 14.8 |
| Rodents | 8 | 13.1 |
| Wild boars | 5 | 8.2 |
| Carnivores | 3 | 4.9 |
| Reptiles | 2 | 3.3 |
| Pangolin | 1 | 1.6 |

**S6. Risk of Bias Assessment Summary**

Each study included in this systematic review was critically appraised for methodological rigor and potential risk of bias using an 11-item checklist specifically adapted from the Joanna Briggs Institute (JBI) tool for prevalence studies and modified to reflect ecological and One Health aspects of AMR surveillance in wildlife.

The following domains were evaluated:

1. Whether the study clearly stated its objectives and specified the intended role of wildlife (e.g., sentinel, vector, or victim) in AMR dynamics.
2. Representativeness of the sampling frame for the target wildlife population.
3. Clarity and appropriateness of the sampling strategy (random, stratified, systematic, or justified convenience).
4. Adequacy of site description (coordinates, habitat type, and degree of anthropogenic exposure).
5. Justification of the sample size based on expected prevalence or population size.
6. Justification of the choice of wildlife species or taxon sampled.
7. Inclusion of relevant ecological or behavioural covariates (e.g., diet, ranging pattern, habitat, mobility, or human interface).
8. Description of field collection, transport, and contamination-control procedures.
9. Standardization and reporting of bacterial identification and antimicrobial susceptibility testing (AST) methods (e.g., CLSI, EUCAST).
10. Clarity and standardization of antimicrobial resistance (AMR) or multidrug resistance (MDR) definitions where applicable.
11. Transparency in reporting numerators, denominators, and resistance outcomes.

Each criterion was scored as:

- 1 = clearly met
- 0.5 = partially met or unclear
- 0 = not met or not reported

Total possible scores ranged from 0 to 11, with higher values indicating stronger methodological quality and lower risk of bias. No studies were excluded based on quality score alone; however, the appraisal informed the interpretation of heterogeneity, robustness of pooled estimates, and the overall strength of evidence. A detailed summary of individual study scores is presented in Supplementary Table 5.

Supplementary Table 5 Methodological risk of bias appraisal of included studies. Each study was scored using the above framework, reflecting methodological quality and informing the credibility of quantitative syntheses.

| Author | Clear study objectives & wildlife role defined | Representative sampling frame | Sampling strategy adequate & described | Study site adequately described | Sample size justification | Justification for wildlife species selected | Ecological/behavioural data captured | Field methods & contamination-control described | Bacterial ID & AST methods standardised | AMR/MDR definitions clearly stated | Clear numerators, denominators, outcomes | Total score |
| --- | --- | --- | --- | --- | --- | --- | --- | --- | --- | --- | --- | --- |
| Routmann et al | 0 | 0 | 0 | 0 | 0 | 0 | 0 | 1 | 1 | 1 | 1 | **4** |
| Obodoechi et al | 1 | 1 | 0 | 1 | 1 | 1 | 1 | 1 | 1 | 1 | 1 | **10** |
| Gakuya et al | 1 | 0 | 0 | 0 | 1 | 0 | 0 | 1 | 0 | 0 | 1 | **4** |
| Adade et al | 1 | 0 | 0 | 1 | 0 | 1 | 0 | 1 | 1 | 0 | 1 | **6** |
| Klee et al | 0 | 0 | 0 | 0.5 | 0 | 0.5 | 0 | 1 | 1 | 0 | 0 | **3** |
| Nguema et al | 1 | 0 | 0 | 1 | 0 | 1 | 1 | 1 | 1 | 0 | 1 | **7** |
| Mubita et al | 0 | 0 | 0 | 0.5 | 0 | 0 | 0 | 0 | 1 | 0 | 1 | **2.5** |
| Schaufler et al | 1 | 0 | 1 | 1 | 0 | 1 | 1 | 1 | 1 | 0 | 1 | **8** |
| Ahmed et al | 1 | 0 | 0 | 0 | 0 | 1 | 1 | 0.5 | 1 | 0 | 1 | **5.5** |
| 1- Bachiri et al | 0 | 0 | 0 | 0.5 | 0 | 1 | 0 | 1 | 0 | 0 | 0 | **2.5** |
| Jobbi ns & Alexander | 1 | 0 | 1 | 1 | 0.5 | 1 | 1 | 1 | 1 | 1 | 1 | **9.5** |
| Nowak et al | 1 | 0 | 0 | 1 | 0 | 1 | 1 | 1 | 1 | 0 | 1 | **7** |
| Kabali et al | 1 | 0 | 0 | 1 | 0 | 1 | 1 | 1 | 1 | 1 | 1 | **8** |
| Albrechtova et al | 1 | 1 | 0 | 1 | 0 | 1 | 0 | 1 | 1 | 0 | 1 | **7** |
| Adesoji et al | 1 | 0 | 0 | 1 | 0 | 1 | 0 | 1 | 1 | 0 | 1 | **6** |
| Bachiri et al | 1 | 0 | 0 | 1 | 0 | 0 | 0 | 1 | 1 | 0 | 1 | **5** |
| Gharout-Sait et al | 0 | 0 | 0 | 0 | 0 | 0 | 0 | 0 | 1 | 1 | 1 | **3** |
| Yinda et al | 1 | 0 | 0 | 1 | 0 | 1 | 0 | 1 | 1 | 1 | 1 | **7** |
| Qiao et al | 0 | 0 | 0 | 1 | 0 | 1 | 0.5 | 1 | 1 | 0 | 1 | **5.5** |
| Abdallah et al | 0 | 1 | 0 | 1 | 0 | 1 | 0.5 | 1 | 1 | 1 | 1 | **7.5** |
| Olatimehin et al | 0 | 0 | 1 | 1 | 0 | 1 | 0 | 0 | 1 | 0 | 1 | **5** |
| Eze et al | 1 | 0 | 0 | 0 | 0 | 0 | 0 | 0 | 0.5 | 0 | 1 | **2.5** |
| Glover et al | 0 | 1 | 1 | 1 | 0 | 1 | 1 | 1 | 1 | 1 | 1 | **9** |
| Pesapane et al | 1 | 1 | 0.5 | 1 | 0 | 1 | 1 | 1 | 1 | 1 | 1 | **9.5** |
| Loucif et al | 0 | 0 | 0 | 1 | 0 | 0 | 1 | 1 | 1 | 1 | 0.5 | **5.5** |
| Said et al | 1 | 0 | 0 | 0 | 0 | 1 | 0 | 1 | 1 | 1 | 1 | **6** |
| Rwego et al | 1 | 1 | 1 | 1 | 0 | 1 | 1 | 1 | 1 | 0 | 0 | **8** |
| Yahia et al | 1 | 0 | 0 | 1 | 0 | 1 | 0 | 1 | 1 | 1 | 1 | **7** |
| Yahia et al | 1 | 0 | 0 | 1 | 0 | 1 | 0 | 0 | 1 | 1 | 1 | **6** |
| Benavides et al | 1 | 1 | 1 | 1 | 1 | 0 | 1 | 0 | 1 | 0.5 | 1 | **8.5** |
| Katakweba et al | 1 | 1 | 1 | 1 | 0 | 1 | 1 | 1 | 1 | 0 | 1 | **9** |
| Kipkorir et al | 1 | 0 | 0 | 0 | 0 | 1 | 0 | 0 | 1 | 1 | 1 | **5** |
| Foster-Nyarko et al | 1 | 1 | 1 | 1 | 0 | 1 | 0 | 1 | 1 | 1 | 1 | **9** |
| Wilson et al | 1 | 0 | 0 | 1 | 0 | 1 | 0 | 1 | 1 | 1 | 1 | **7** |
| Wiethoff et al | 1 | 0 | 0 | 1 | 0 | 1 | 0 | 1 | 1 | 1 | 1 | **7** |
| Janatova et al | 1 | 1 | 0 | 1 | 0 | 1 | 1 | 0 | 1 | 1 | 1 | **8** |
| Okunlade | 1 | 0 | 0 | 1 | 0 | 1 | 0 | 0.5 | 1 | 1 | 1 | **6.5** |
| More et al | 1 | 1 | 1 | 1 | 0 | 1 | 0 | 1 | 1 | 1 | 1 | **9** |
| Nguema et al | 1 | 1 | 0 | 0 | 0 | 0 | 0 | 1 | 1 | 1 | 1 | **6** |
| Nguema et al | 0 | 0 | 0 | 0 | 0 | 1 | 0 | 1 | 0.5 | 1 | 1 | **4.5** |
| Hassell et al | 1 | 1 | 1 | 1 | 0 | 1 | 1 | 1 | 1 | 1 | 1 | **10** |
| Weiss et al | 1 | 0 | 0 | 1 | 0 | 1 | 0.5 | 1 | 1 | 1 | 1 | **7.5** |
| King & Schmidt | 1 | 0 | 0 | 0 | 0 | 1 | 0 | 0.5 | 1 | 1 | 1 | **5.5** |
| Clausen & Ashford | 0 | 0 | 0 | 0 | 0 | 0 | 0 | 1 | 1 | 1 | 1 | **4** |
| Fadel et al | 1 | 0 | 0 | 0 | 0 | 0 | 0 | 0.5 | 1 | 1 | 1 | **4.5** |
| Bellil et al | 1 | 0 | 0 | 0 | 0 | 0 | 0 | 0.5 | 0.5 | 1 | 1 | **4** |
| Brisson et al | 1 | 0 | 0 | 1 | 0 | 1 | 0.5 | 1 | 1 | 1 | 1 | **7.5** |
| Klibi et al | 1 | 0 | 0 | 0 | 0 | 0 | 0 | 0.5 | 0.5 | 1 | 1 | **4** |
| Schaumburg et al | 0 | 0 | 0 | 0 | 0 | 1 | 0 | 0 | 1 | 1 | 1 | **4** |
| Mairi et al | 1 | 1 | 0 | 0 | 0 | 1 | 0 | 1 | 1 | 1 | 1 | **7** |
| Eidaroos et al | 0 | 0 | 0 | 0 | 0 | 0 | 0 | 1 | 1 | 1 | 1 | **4** |
| Fashae et al | 1 | 1 | 1 | 1 | 0 | 1 | 0 | 1 | 1 | 1 | 1 | **9** |
| Selmi et al | 1 | 1 | 1 | 0 | 0 | 1 | 0 | 1 | 1 | 1 | 1 | **8** |
| Modupe et al | 1 | 1 | 1 | 1 | 0 | 1 | 1 | 1 | 1 | 1 | 1 | **10** |
| Shin et al | 0 | 0 | 0 | 0 | 0 | 0 | 0 | 1 | 1 | 1 | 1 | **4** |
| Akobi et al | 1 | 0 | 0 | 0 | 0 | 1 | 0 | 0.5 | 1 | 1 | 1 | **5.5** |
| Nguema et al | 1 | 0 | 0 | 1 | 0 | 1 | 0 | 0.5 | 1 | 1 | 1 | **6.5** |
| Sonoal et al | 1 | 1 | 1 | 1 | 0 | 1 | 0 | 1 | 1 | 1 | 1 | **9** |
| Tayh et al | 1 | 1 | 0 | 0 | 0 | 1 | 0 | 0.5 | 1 | 1 | 1 | **6.5** |
| Hachem et al | 1 | 1 | 0 | 0 | 0 | 1 | 0 | 1 | 1 | 1 | 1 | **7** |
| Raheem et al | 1 | 1 | 0 | 0 | 0 | 1 | 0 | 0.5 | 1 | 1 | 1 | **6.5** |

**Supplementary note 7. Multidrug resistance**

MDR definitions were not reclassified in this review but were recorded as reported by the original authors. Most studies referenced or followed the criteria outlined by Magiorakos et al. (2012), while a few applied broader or study-specific interpretations (e.g., resistance to ≥2 antibiotic classes). This table provides an overview of definitional variability across studies.

Supplementary Table 6 Definitions and reported prevalence of multidrug resistance (MDR) among bacterial isolates from African wildlife. Most studies followed the criteria by Magiorakos et al. (2012 while a few applied broader definitions (e.g., ≥2 classes).

| Author | # MDR isolates | Proportion | MDR Definition |
| --- | --- | --- | --- |
| Obodoechi et al | 8 | 22.9% | Magiorakos et al; resistance to at least one antimicrobial agent in three or more antimicrobial classes |
| Yinda et al | 12 | 18.8% | MDR as bacteria that are resistant to three or more classes of antimicrobial agents |
| Foster-Nyarko et al | 3 | 3.0% | Magiorakos et al; resistance to three or more antimicrobial classes |
| Wilson et al | 3 | 3.0% | Magiorakos et al; isolates resistant to two, three, four, five, six and seven antimicrobial categories |
| Hassell et al | 252 |  | Magiorakos et al; non-susceptibility to ≥1 agent in ≥3 classes; also assessed ≥7-class and pan-resistance levels |
| King & Schmidt | 14 | 46.7% | Magiorakos; resistance to antibiotics from at least three different classes |
| Mairi et al | 4 | 36.4% | Magiorakos et al; resistance to at least one agent in three or more antimicrobial classes |
| Fashae et al | 77 | 83.7% | resistance to three or more antimicrobial classes |
| Selmi et al | 57 | 55.9% | Magiorakos et al. All strains showing non-susceptibility patterns to more than 3 antimicrobial-classes were considered as multi-resistant |
| Sonola et al | 15 | 26.8% | Magiorakos et al; non-susceptible to three or more drugs from different classes of antibiotics |
| Tayh et al | 5 | 41.7% | Magiorakos et al; isolate exhibiting resistance to at least one compound from three or more antimicrobial classes |
| Hachem et al | 51 | 55.4% | resistance to three or more antimicrobial classes |
| Raheem et al | 3 | 27.0% | Magiorakos et al. (MDR- non-susceptibility to at least one agent in three or more antimicrobial categories; XDR- non-susceptibility to at least one agent in all but two or fewer antimicrobial categories; PDR- non-susceptibility to all all agents in all antimicrobial categories (no agents tested as susceptible)) |

**Supplementary note 8. Reporting Limitations in Antibiotic Class–Specific Data**

Quantitative meta-analysis by antibiotic class was not possible due to the inconsistent and incomplete reporting of antibiotic-specific resistance data across studies. Majority of studies reported overall antimicrobial resistance (AMR) proportions without providing class-specific numerators and denominators, while others presented results qualitatively as “resistant” or “susceptible” without specifying the number of isolates tested. In some cases, multiple antibiotics within a class were inconsistently tested or aggregated under broad resistance categories, further complicating comparability. As a result, statistical synthesis by antibiotic class would have introduced analytical bias and misrepresented the underlying data. To illustrate this heterogeneity, we have summarised below a sample of the range of antibiotic classes and agents tested across studies, highlighting the variable reporting formats and limited quantitative detail that precluded pooled analysis.

Supplementary Table 7. Antibiotic classes and specific agents tested in studies reporting AMR in Escherichia coli from African wildlife. Heterogeneous reporting formats and lack of denominators across studies precluded quantitative class-level meta-analysis.

| author | Host | organism | %AMR | β-lactams | | | | | | Aminoglycosides | | | | Fluoroquinolone | | Carbapenems | | |
| --- | --- | --- | --- | --- | --- | --- | --- | --- | --- | --- | --- | --- | --- | --- | --- | --- | --- | --- |
|  |  |  |  | **Amoxicillin** | **Ampicillin** | **Augmentin** | **Cefepime** | **Cefotaxime** | **Ceftazidime** | **Amikacin** | **Gentamicin** | **Streptomycin** | **Tobramycin** | **Ciprofloxacin** | **Nalidixic Acid** | **Ertapenem** | **Imipenem** | **Meropenem** |
| Routman et al | NHP | E. coli | 17.60% |  | 9% |  |  |  |  |  |  | 10.4 |  |  |  |  |  |  |
| Said et al |  | E. coli |  |  | 3% |  |  | 3% | 3% | 0% | 0% |  | 0% | 47% |  |  | 0% |  |
| 1- Bachiri et al | NHP | E. coli | 100% | R |  | Resistant | Resistant | Resistant |  | Susceptible |  |  | Resistant | Resistant | Resistant | Resistant | Susceptible |  |
| Nowak et al | Bats | E. coli | 89.70% |  | 0 | 0 |  | 0 | 0 |  | 0 |  |  | 0 | 0 |  |  |  |
| Bachiri et al | Herbivores | E. coli | 1 | R |  |  | Susceptible | Resistant | Susceptible | Susceptible | Susceptible |  | Resistant | Susceptible | Susceptible | Resistant | Susceptible | Resistant |
| Katakweba et al | Herbivores | E. coli | 47.60% |  | 47.60% | 11.90% |  | 11.90% |  |  | 23.8 |  |  |  |  |  |  |  |
| Kipkorir et al | Herbivores | E. coli | 100% |  | 100 | 73.3 |  |  |  |  | 20 |  |  |  |  |  |  |  |
| Okunlade | NHP | E. coli | 84% |  | Susceptible | Resistant | Susceptible |  | Susceptible | Susceptible | Susceptible |  | Susceptible |  |  | Susceptible |  | Susceptible |
| King & Schmidt | Herbivores | E. coli | 90% |  | Susceptible | Resistant |  | Resistant | Resistant |  | Resistant |  | Resistant | Susceptible |  | Resistant |  | Susceptible |
| King & Schmidt | Herbivores | E. coli | 43.30% |  | Susceptible | Resistant |  | Susceptible | Resistant |  | Resistant |  | Resistant | Susceptible |  | Susceptible |  | Susceptible |
| Brisson et al | Herbivores | E. coli | 24.10% | 53 |  |  |  |  | 28 |  |  | 37 |  | 4 |  |  |  |  |
| Fashae et al | Wild birds | E. coli | 98% |  | Susceptible |  |  | Resistant |  |  | Resistant | Resistant |  | Resistant |  | Susceptible | Susceptible |  |

**Supplementary note 9. Subgroup Meta-analyses**

To explore variation in antimicrobial resistance (AMR) prevalence across key wildlife host groups, we conducted subgroup meta-analyses for herbivores, non-human primates, and wild birds. Forest and funnel plots are provided for each subgroup, summarising pooled estimates, heterogeneity metrics, and potential small-study effects based on logit-transformed proportions.

**Plot 9.1; AMR in African wildlife**


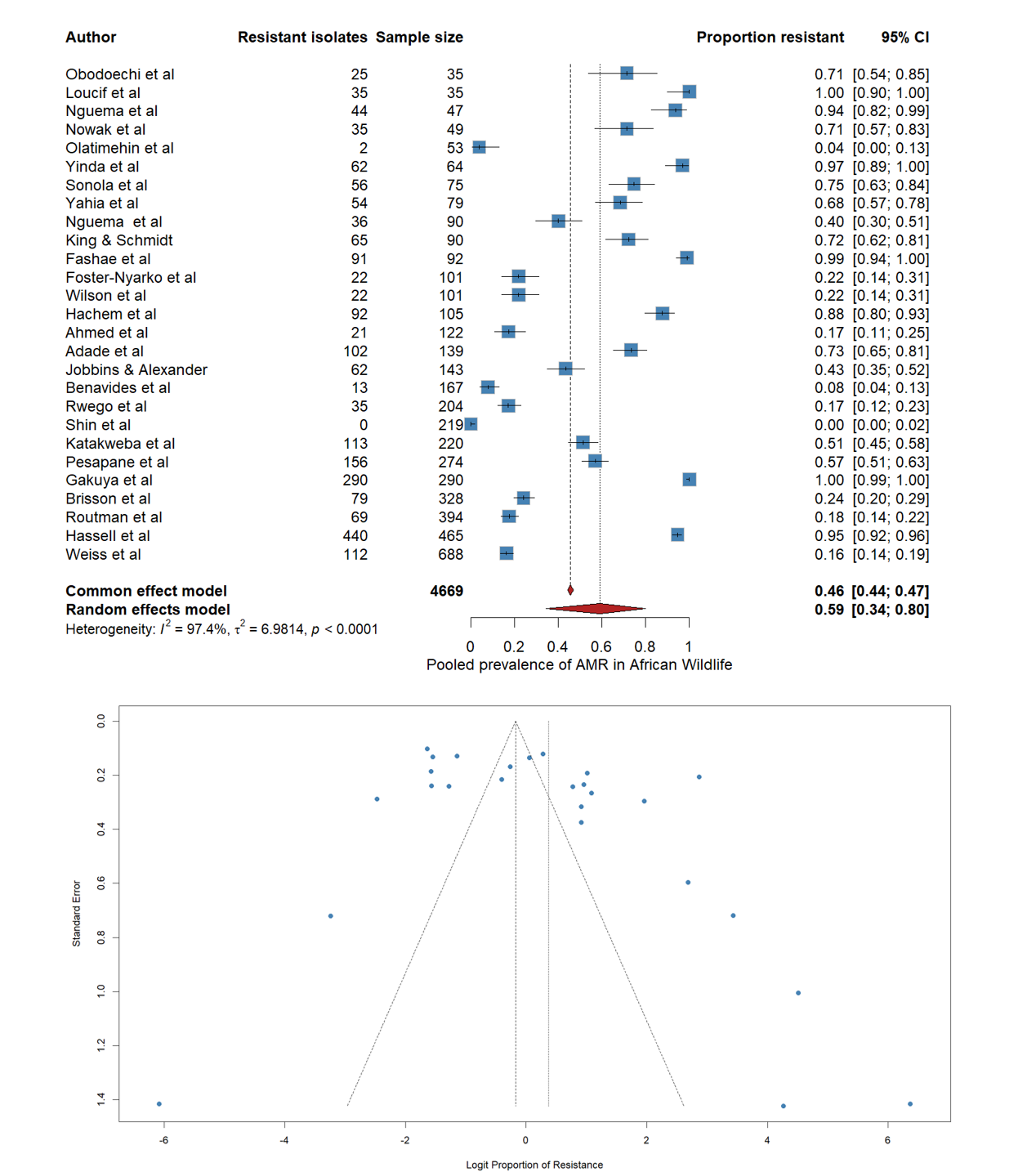


Supplementary Figure 2 Pooled prevalence and publication bias assessment of antimicrobial resistance (AMR) in African wildlife.
The upper panel presents a forest plot of studies (n = 4,669 bacterial isolates) reporting phenotypic AMR prevalence in African wildlife. Each blue square represents a study-specific estimate, with horizontal lines showing 95% confidence intervals; the red diamond indicates the pooled prevalence under a random-effects model. The pooled AMR prevalence was 59% (95% CI: 34–80%), with substantial heterogeneity (I² = 96.6%). The lower panel shows a funnel plot of logit-transformed resistance proportions against their standard errors, used to evaluate potential small-study effects.

**Plot 9.2; Handling Heterogeneity**

To assess the influence of individual studies on the overall pooled prevalence, we performed a leave-one-out (LOO) sensitivity test (Supplementary Figure 3). Sequential omission of each study produced minimal variation in the pooled prevalence of AMR which remained between 59% and 71% under the random effects model (overall 65%; 95%CI: 38**–**85%, I^2^≈ 96.6%). The values showed negligible change upon exclusion of any single study, indicating that no individual study disproportionately influenced the summary estimate.


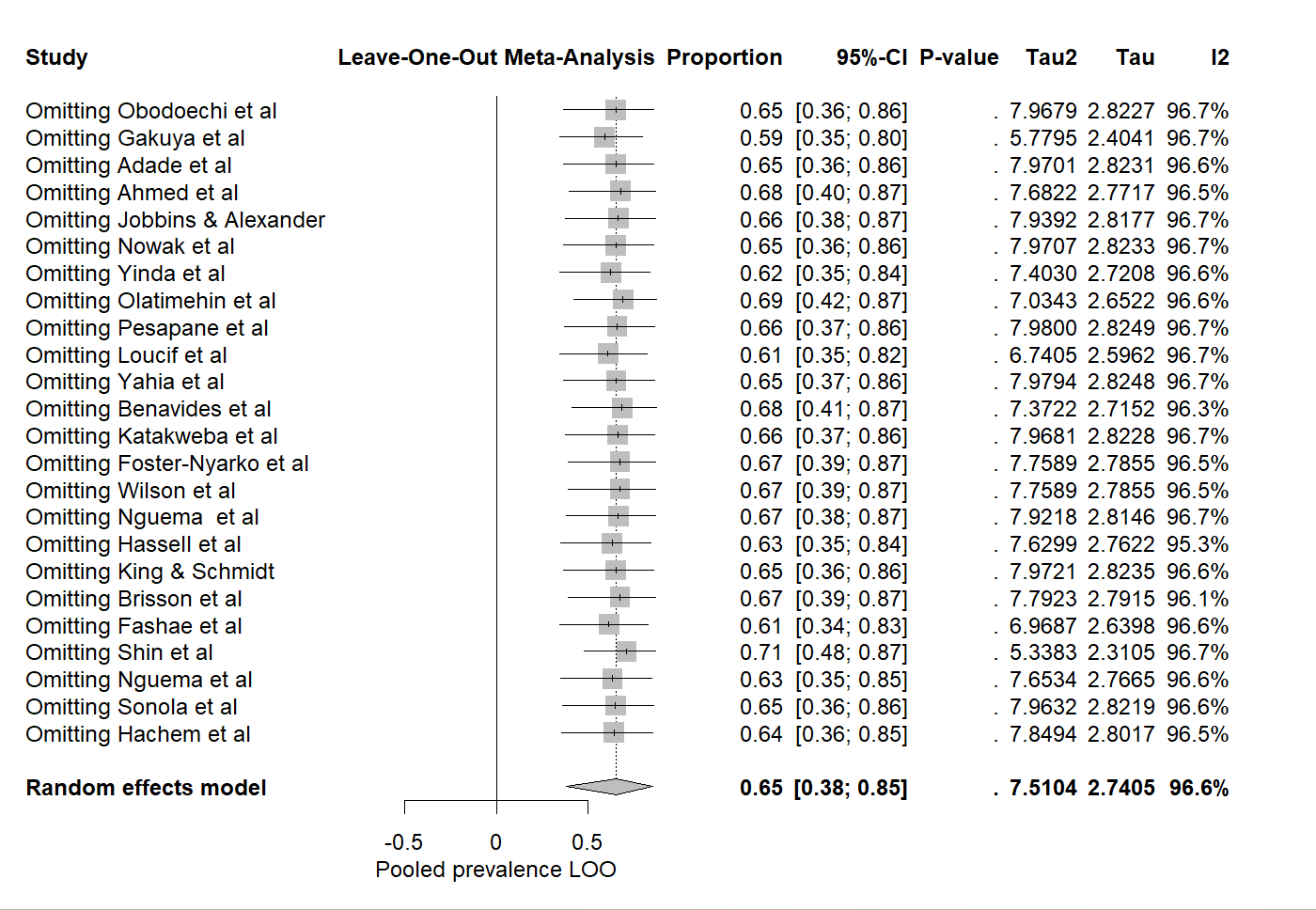


Supplementary Figure 3. Leave-one-out (LOO) sensitivity analysis showing the influence of each study on the pooled prevalence of antimicrobial resistance (AMR) in African wildlife. Each row represents the recalculated pooled prevalence after excluding one study at a time. Grey squares indicate point estimates, horizontal lines denote 95% confidence intervals, and the diamond represents the overall random-effects model estimate.

**Plot 9.3; AMR in Herbivores
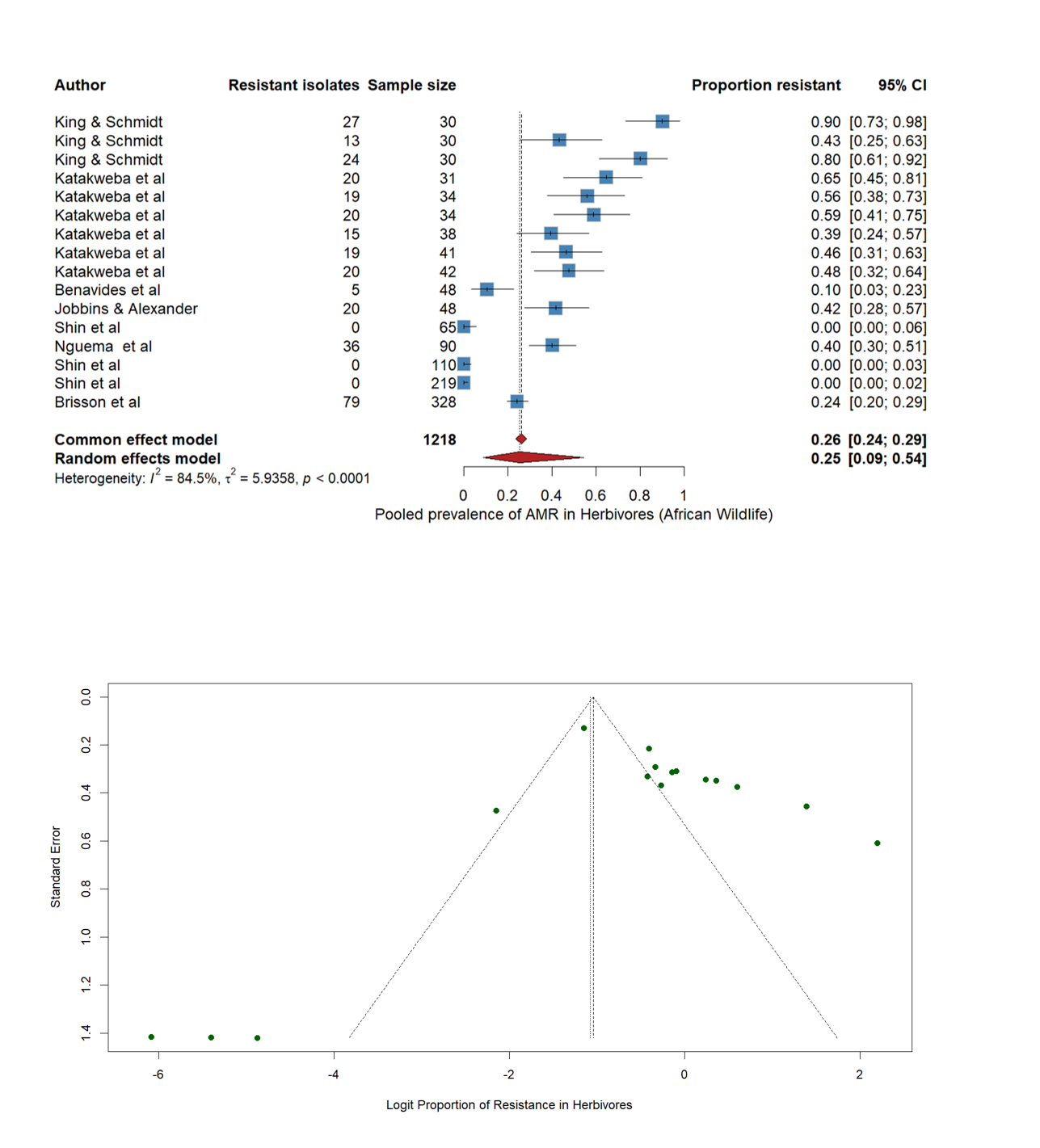
**

Supplementary Figure 4 Pooled prevalence and publication bias assessment of antimicrobial resistance (AMR) in herbivores in Africa.
The forest plot (top) displays individual and pooled estimates of AMR prevalence from studies on herbivorous wildlife species, 1,218 bacterial isolates. The pooled prevalence under a random-effects model was 25% (95% CI: 9%–54%), with substantial heterogeneity (I² = 84.5%). Each square represents a study-specific estimate, with horizontal lines indicating 95% confidence intervals. The funnel plot (bottom) presents logit-transformed resistance proportions plotted against their standard errors to assess asymmetry and small-study effects.

**Plot 9.4; AMR in Non-human primates
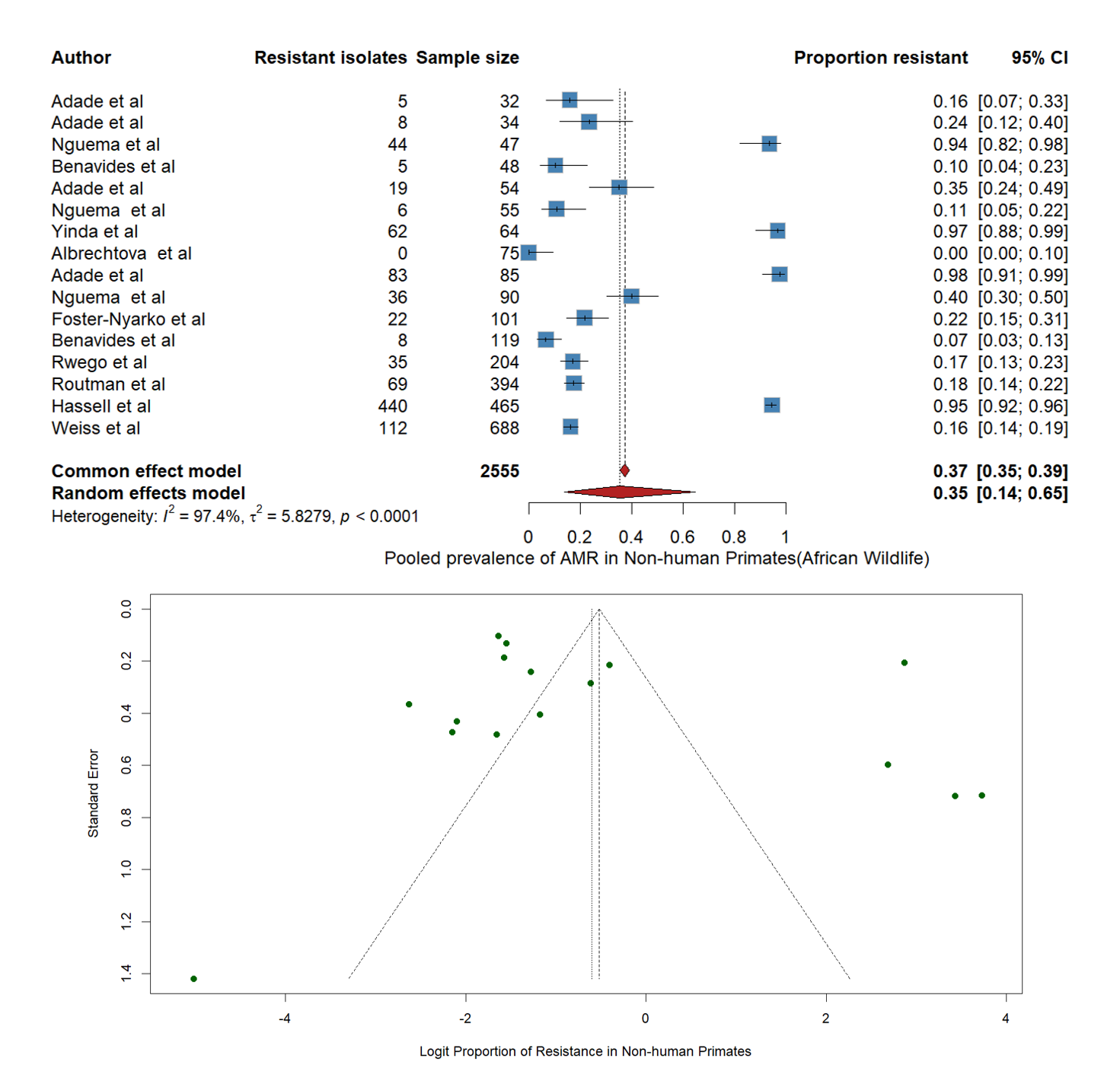
**

Supplementary Figure 5 Pooled prevalence and publication bias assessment of antimicrobial resistance (AMR) in non-human primates in Africa.
The forest plot (top) summarises AMR prevalence estimates from studies in non-human primates. The pooled prevalence under a random-effects model was 35% (95% CI: 14%–65%) across 2,555 isolates. Each study's point estimate and 95% confidence interval are shown, with substantial heterogeneity observed (I² = 97.4%). The funnel plot (bottom) presents logit-transformed resistance proportions plotted against their standard errors to assess small-study effects.

**Plot 9.5; AMR in Wild birds**


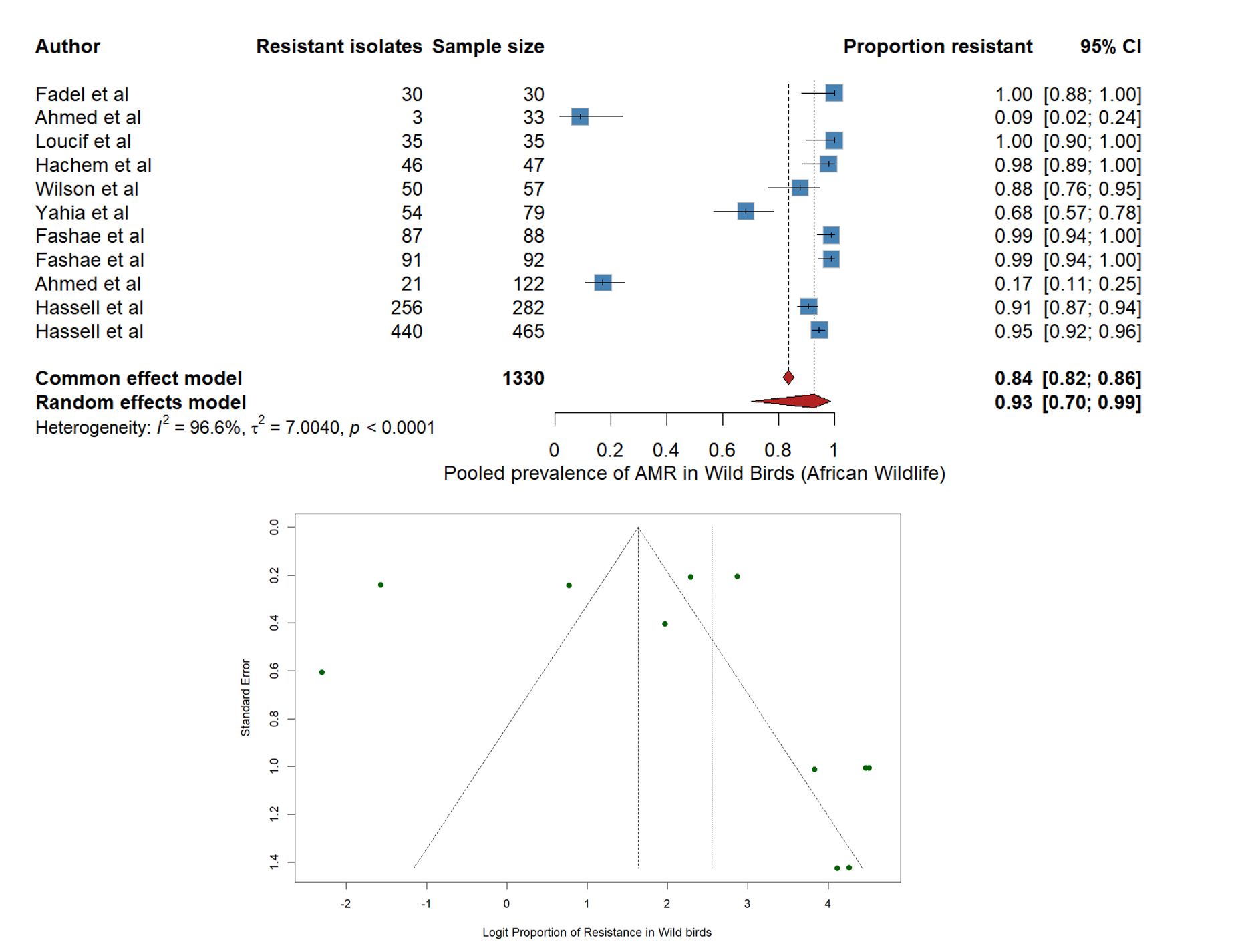


Supplementary Figure 6 Pooled prevalence and publication bias assessment of antimicrobial resistance (AMR) in wild birds in Africa.
The upper panel displays a forest plot of studies reporting phenotypic resistance in wild birds, showing individual study estimates and 95% confidence intervals. The pooled prevalence of AMR using a random-effects model was 93% (95% CI: 70%–99%) based on 1,330 isolates. The lower panel shows a funnel plot of logit-transformed resistance proportions against their standard errors, used to evaluate potential small-study effects.

**References**

1. Bachiri, T. *et al.* First Report of the Plasmid-Mediated Colistin Resistance Gene mcr-1 in Escherichia coli ST405 Isolated from Wildlife in Bejaia, Algeria. *Microbial drug resistance (Larchmont, N.Y.)* **24**, 890–895 (2018).

2. Bachiri, T. *et al.* Occurrence of Carbapenemase-Producing Enterobacteriaceae Isolates in the Wildlife: First Report of OXA-48 in Wild Boars in Algeria. *MICROBIAL DRUG RESISTANCE* **24**, 337–345 (2018).

3. Bellil, Z. *et al.* Comprehensive Genomic Characterization of Antibiotic Resistance, Virulence, and Clonality in Salmonella Isolates from Wild Animals in Algeria. *EcoHealth* **20**, 343–348 (2023).

4. Gharout-Sait, A. *et al.* Occurrence of Carbapenemase-Producing Klebsiella pneumoniae in Bat Guano. *MICROBIAL DRUG RESISTANCE* **25**, 1057–1062 (2019).

5. Hachem, Y. *et al.* Phenotypic and molecular characterization of vancomycin resistant enterococci from wild birds: first detection of a plasmid-borne vanC1 in Enterococcus faecalis. *LETTERS IN APPLIED MICROBIOLOGY* **77**, (2024).

6. Loucif, L. *et al.* Detection of blaOXA-48 and mcr-1 Genes in Escherichia coli Isolates from Pigeon (Columba livia) in Algeria. *Microorganisms* **10**, (2022).

7. Mairi, A. *et al.* First Report of CC5-MRSA-IV-SCCfus ‘Maltese Clone’ in Bat Guano. *Microorganisms* **9**, (2021).

8. Jobbins, S. E. & Alexander, K. A. FROM WHENCE THEY CAME—ANTIBIOTIC-RESISTANT ESCHERICHIA COLI IN AFRICAN WILDLIFE. *Journal of Wildlife Diseases* **51**, 811–820 (2015).

9. Pesapane, R., Ponder, M. & Alexander, K. A. Tracking pathogen transmission at the human-wildlife interface: banded mongoose and Escherichia coli. *EcoHealth* **10**, 115–128 (2013).

10. Janatova, M. *et al.* Antimicrobial-resistant Enterobacteriaceae from humans and wildlife in Dzanga-Sangha Protected Area, Central African Republic. *Veterinary microbiology* **171**, 422–431 (2014).

11. Albrechtova, K. *et al.* Low rates of antimicrobial-resistant Enterobacteriaceae in wildlife in Tai National Park, Cote d’Ivoire, surrounded by villages with high prevalence of multiresistant ESBL-producing Escherichia coli in people and domestic animals. *PloS one* **9**, e113548–e113548 (2014).

12. Klee, S. R. *et al.* Characterization of Bacillus anthracis-like bacteria isolated from wild great apes from Cote d’Ivoire and Cameroon. *Journal of bacteriology* **188**, 5333–5344 (2006).

13. Nowak, K. *et al.* Highly diverse and antimicrobial susceptible Escherichia coli display a naive bacterial population in fruit bats from the Republic of Congo. *PLOS ONE* **12**, (2017).

14. Ahmed, Z. S., Elshafiee, E. A., Khalefa, H. S., Kadry, M. & Hamza, D. A. Evidence of colistin resistance genes (mcr-1 and mcr-2) in wild birds and its public health implication in Egypt. *ANTIMICROBIAL RESISTANCE AND INFECTION CONTROL* **8**, (2019).

15. Eidaroos, N. H., Youssef, A., El-Sebae, A., Enany, M. E. & Farid, D. S. Genotyping of enterotoxigenic methicillin-resistant Staphylococcus aureus (MRSA) and vancomycin-resistant Staphylococcus aureus (VRSA) among commensal rodents in North Sinai, Egypt. *JOURNAL OF APPLIED MICROBIOLOGY* **132**, 2331–2341 (2022).

16. Fadel, H. M., Afifi, R. & Al-Qabili, D. M. Characterization and zoonotic impact of Shiga toxin producing Escherichia coli in some wild bird species. *VETERINARY WORLD* **10**, 1118–1128 (2017).

17. Abdel-Raheem, S. M., Al-Sultan, S. I. & El-Tarabili, R. M. First Detection of Vibrio parahaemolyticus in Migratory Birds in Egypt: Antibiogram, Virulence, and Resistance Gene Profiles Indicating Zoonotic and Public Health Risks. *Current Microbiology* **82**, 15-Article No.: 15 (2025).

18. Mbehang Nguema, P. P. *et al.* Isolation of multiple drug-resistant enteric bacteria from feces of wild Western Lowland Gorilla (Gorilla gorilla gorilla) in Gabon. *The Journal of veterinary medical science* **77**, 619–623 (2015).

19. Nguema, P. P. M., Tsuchida, S. & Ushida, K. Bacteria culturing and isolation under field conditions of Moukalaba-Doudou National Park, Gabon, and preliminary survey on bacteria carrying antibiotic resistance genes. *TROPICS* **23**, 165–174 (2015).

20. Nguema, P. P. M. *et al.* High level of intrinsic phenotypic antimicrobial resistance in enterobacteria from terrestrial wildlife in Gabonese national parks. *PLOS ONE* **16**, (2021).

21. Nguema, P. P. M. *et al.* Characterization of ESBL-Producing Enterobacteria from Fruit Bats in an Unprotected Area of Makokou, Gabon. *MICROORGANISMS* **8**, (2020).

22. Wiethoff, J. P. *et al.* Pharyngeal Communities and Antimicrobial Resistance in Pangolins in Gabon. *Microbiology spectrum* **11**, e0066423–e0066423 (2023).

23. Yinda, L. *et al.* Phylogenetic Groups, Pathotypes and Antimicrobial Resistance of Escherichia coli Isolated from Western Lowland Gorilla Faeces (Gorilla gorilla gorilla) of Moukalaba-Doudou National Park (MDNP). *PATHOGENS* **11**, (2022).

24. Benavides, J. A., Salgado-Caxito, M., Torres, C. & Godreuil, S. Public Health Implications of Antimicrobial Resistance in Wildlife at the One Health Interface. *Medical Sciences Forum* **25**, 1 (2024).

25. Foster-Nyarko, E. *et al.* Genomic diversity of Escherichia coli isolates from non-human primates in the Gambia. *Microbial genomics* **6**, (2020).

26. Adade, E. *et al.* Antimicrobial susceptibility profile of oral and rectal microbiota of non-human primate species in Ghana: A threat to human health. *Veterinary medicine and science* https://doi.org/10.1002/vms3.1271 (2023) doi:10.1002/vms3.1271.

27. Modupe, S. L. *et al.* Protected but not from Contamination: Antimicrobial Resistance Profiles of Bacteria from Birds in a Ghanaian Forest Protected Area. *ENVIRONMENTAL HEALTH INSIGHTS* **15**, (2021).

28. Schaufler, K. *et al.* Clinically Relevant ESBL-Producing K. pneumoniae ST307 and E. coli ST38 in an Urban West African Rat Population. *FRONTIERS IN MICROBIOLOGY* **9**, (2018).

29. Gakuya, F. M., Kyule, M. N., Gathura, P. B. & Kariuki, S. Antimicrobial resistance of bacterial organisms isolated from rats. *East African medical journal* **78**, 646–649 (2001).

30. Hassell, J. M. *et al.* Clinically relevant antimicrobial resistance at the wildlife-livestock-human interface in Nairobi: an epidemiological study. *The Lancet. Planetary health* **3**, e259–e269 (2019).

31. Kipkorir, K. C., Ang’ienda, P. O., Onyango, D. M. & Onyango, P. O. Antibiotic Resistance of Escherichia coli from Humans and Black Rhinoceroses in Kenya. *EcoHealth* **17**, 41–51 (2020).

32. Clausen, B. & Ashford, W. A. Bacteriologic survey of black rhinoceros (Diceros bicornis). *Journal of wildlife diseases* **16**, 475–480 (1980).

33. Qiao, N. *et al.* Physiological and genomic characterization of Lactiplantibacillus plantarum isolated from Indri indri in Madagascar. *Journal of applied microbiology* **134**, (2023).

34. Adesoji, T. O. *et al.* Molecular characterization of non-aureus staphylococci and *Mammaliicoccus* from Hipposideros bats in Southwest Nigeria. *SCIENTIFIC REPORTS* **14**, (2024).

35. Akobi, B., Aboderin, O., Sasaki, T. & Shittu, A. Characterization of Staphylococcus aureus isolates from faecal samples of the Straw-Coloured Fruit Bat (Eidolon helvum) in Obafemi Awolowo University (OAU), Nigeria. *BMC microbiology* **12**, 279–279 (2012).

36. Eze, E. A., Mustapha, K. J., Ndubuisi, I. A., Nwodo, U. & Okoh, A. Studies on Drug Resistance among Klebsiella and Citrobacter spp Isolated from two Human Groups and Wild Animals. *JUNDISHAPUR JOURNAL OF MICROBIOLOGY* **11**, (2018).

37. Fashae, K., Engelmann, I., Monecke, S., Braun, S. D. & Ehricht, R. Molecular characterisation of extended-spectrum s-lactamase producing Escherichia coli in wild birds and cattle, Ibadan, Nigeria. *BMC veterinary research* **17**, 33–33 (2021).

38. Obodoechi, L. O. *et al.* Antimicrobial resistance in Escherichia coli isolates from frugivorous (Eidolon helvum) and insectivorous (Nycteris hispida) bats in Southeast Nigeria, with detection of CTX-M-15 producing isolates. *Comparative immunology, microbiology and infectious diseases* **75**, 101613–101613 (2021).

39. Okunlade, A. O. *et al.* Quinolone resistance markers in fluoroquinolone-resistant, non-ESBL- producing Escherichia coli isolated from non-human primates at selected zoological gardens and tourist centres. *International Journal of Environmental Studies* **80**, 687–698 (2023).

40. Olatimehin, A. *et al.* Staphylococcus aureus Complex in the Straw-Colored Fruit Bat (Eidolon helvum) in Nigeria. *FRONTIERS IN MICROBIOLOGY* **9**, (2018).

41. Abdallah, R. *et al.* Population Diversity of Antibiotic Resistant Enterobacterales in Samples From Wildlife Origin in Senegal: Identification of a Multidrug Resistance Transposon Carrying blaCTX–M–15 in Escherichia coli. *Frontiers in Microbiology* **13**, (2022).

42. Glover, B., Wentzel, J., Jenkins, A. & Van Vuuren, M. The first report of Escherichia fergusonii isolated from non-human primates, in Africa. *ONE HEALTH* **3**, 70–75 (2017).

43. King, T. L. B. & Schmidt, S. Assessment of three indigenous South African herbivores as potential reservoirs and vectors of antibiotic-resistant Escherichia coli. *EUROPEAN JOURNAL OF WILDLIFE RESEARCH* **63**, (2017).

44. More, E. *et al.* Seabirds (Laridae) as a source of Campylobacter spp., Salmonella spp. and antimicrobial resistance in South Africa. *Environmental Microbiology* **19**, 4164–4176 (2017).

45. Wilson, T. K., Zishiri, O. T. & El Zowalaty, M. E. Molecular detection of multidrug and methicillin resistance in Staphylococcus aureus isolated from wild pigeons (Columba livia) in South Africa. *ONE HEALTH* **18**, (2024).

46. Katakweba, A. A. S. *et al.* Antimicrobial resistance in faecal samples from buffalo, wildebeest and zebra grazing together with and without cattle in Tanzania. *Journal of applied microbiology* **118**, 966–975 (2015).

47. PHILLIPS-CONROY, J. E., ROUTMAN, E. J. & MILLER, R. D. ANTIBIOTIC RESISTANCE PATTERNS AND POPULATION STRUCTURE OF ESCHERICHIA-COLI IN HUMAN AND NONHUMAN ASSOCIATED YELLOW BABOONS FROM MIKUMI NATIONAL PARK TANZANIA. *American Journal of Physical Anthropology* **66**, 214–215 (1985).

48. Shin, E., Mduma, S., Keyyu, J., Fyumagwa, R. & Lee, Y. An Investigation of Enterococcus Species Isolated from the African Buffalo (Syncerus caffer) in Serengeti National Park, Tanzania. *Microbes and environments* **32**, 402–406 (2017).

49. Sonola, V. S., Misinzo, G. & Matee, M. I. Occurrence of Multidrug-Resistant Staphylococcus aureus among Humans, Rodents, Chickens, and Household Soils in Karatu, Northern Tanzania. *INTERNATIONAL JOURNAL OF ENVIRONMENTAL RESEARCH AND PUBLIC HEALTH* **18**, (2021).

50. Klibi, N. *et al.* Diversity of species and antibiotic resistance among fecal enterococci from wild birds in Tunisia. Detection of vanA-containing Enterococcus faecium isolates. *EUROPEAN JOURNAL OF WILDLIFE RESEARCH* **61**, 319–323 (2015).

51. Ben Said, L., Jouini, A., Fliss, I., Torres, C. & Klibi, N. Antimicrobial resistance genes and virulence gene encoding intimin in Escherichia coli and Enterococcus isolated from wild rabbits (Oryctolagus cuniculus) in Tunisia. *Acta veterinaria Hungarica* **67**, 477–488 (2019).

52. Selmi, R. *et al.* Prevalence, risk factors and emergence of extended-spectrum beta-lactamase producing-, carbapenem- and colistin-resistant Enterobacterales isolated from wild boar (Sus scrofa) in Tunisia. *Microbial pathogenesis* **163**, 105385–105385 (2022).

53. Tayh, G. *et al.* Risk for public health of multiresistant Shiga toxin-producing Escherichia coli (STEC) in wild boar (Sus scrofa) in Tunisia. *MICROBIAL PATHOGENESIS* **201**, (2025).

54. Ben Yahia, H. *et al.* Detection of CTX-M-15 harboring Escherichia coli isolated from wild birds in Tunisia. *BMC microbiology* **18**, 26–26 (2018).

55. Ben Yahia, H. *et al.* Antimicrobial resistance and genetic lineages of faecal enterococci of wild birds: Emergence of vanA and vanB2 harbouring Enterococcus faecalis. *International journal of antimicrobial agents* **52**, 936–941 (2018).

56. Rwego, I. B., Isabirye-Basuta, G., Gillespie, T. R. & Goldberg, T. L. Gastrointestinal bacterial transmission among humans, mountain gorillas, and livestock in Bwindi Impenetrable National Park, Uganda. *Conservation biology : the journal of the Society for Conservation Biology* **22**, 1600–1607 (2008).

57. Weiss, D. *et al.* Antibiotic-Resistant Escherichia coli and Class 1 Integrons in Humans, Domestic Animals, and Wild Primates in Rural Uganda. *Applied and environmental microbiology* **84**, (2018).

58. Kabali, E. *et al.* Identification of Escherichia coli and Related Enterobacteriaceae and Examination of Their Phenotypic Antimicrobial Resistance Patterns: A Pilot Study at A Wildlife-Livestock Interface in Lusaka, Zambia. *ANTIBIOTICS-BASEL* **10**, (2021).

59. Mubita, C. M. *et al.* Characterization of non-typhoid Salmonellae isolated from domestic animals and wildlife from selected areas of Zambia. *SCIENTIFIC AFRICAN* **8**, (2020).

60. Schaumburg, F. *et al.* Drug-Resistant Human Staphylococcus Aureus in Sanctuary Apes Pose a Threat to Endangered Wild Ape Populations. *American Journal of Primatology* **74**, 1071–1075 (2012).

61. Brisson, L. *et al.* COMPARING ANTIBIOTIC RESISTANCE IN FREE-RANGING VS. CAPTIVE AFRICAN WILD HERBIVORES. *JOURNAL OF WILDLIFE DISEASES* **59**, 224–233 (2023).
